# Interacting forces of predation and fishing affect species’ maturation size

**DOI:** 10.1002/ece3.6995

**Published:** 2020-12-05

**Authors:** Romain Forestier, Julia L. Blanchard, Kirsty L. Nash, Elizabeth A. Fulton, Craig Johnson, Asta Audzijonyte

**Affiliations:** ^1^ Institute for Marine and Antarctic Studies University of Tasmania Hobart TAS Australia; ^2^ Centre for Marine Socioecology Hobart TAS Australia; ^3^ Commonwealth Scientific and Industrial Research Organisation Hobart TAS Australia

**Keywords:** body size, coexistence, evolution, fisheries, food webs, multi‐species size spectrum model

## Abstract

1. Fishing is a strong selective force and is supposed to select for earlier maturation at smaller body size. However, the extent to which fishing‐induced evolution is shaping ecosystems remains debated. This is in part because it is challenging to disentangle fishing from other selective forces (e.g., size‐structured predation and cannibalism) in complex ecosystems undergoing rapid change.
2. Changes in maturation size from fishing and predation have previously been explored with multi‐species physiologically structured models but assumed separation of ecological and evolutionary timescales. To assess the eco‐evolutionary impact of fishing and predation at the same timescale, we developed a stochastic physiologically size‐structured food‐web model, where new phenotypes are introduced randomly through time enabling dynamic simulation of species' relative maturation sizes under different types of selection pressures.
3. Using the model, we carried out a fully factorial in silico experiment to assess how maturation size would change in the absence and presence of both fishing and predation (including cannibalism). We carried out ten replicate stochastic simulations exposed to all combinations of fishing and predation in a model community of nine interacting fish species ranging in their maximum sizes from 10 g to 100 kg. We visualized and statistically analyzed the results using linear models.
4. The effects of fishing on maturation size depended on whether or not predation was enabled and differed substantially across species. Fishing consistently reduced the maturation sizes of two largest species whether or not predation was enabled and this decrease was seen even at low fishing intensities (*F* = 0.2 per year). In contrast, the maturation sizes of the three smallest species evolved to become smaller through time but this happened regardless of the levels of predation or fishing. For the four medium‐size species, the effect of fishing was highly variable with more species showing significant and larger fishing effects in the presence of predation.
5. Ultimately our results suggest that the interactive effects of predation and fishing can have marked effects on species' maturation sizes, but that, at least for the largest species, predation does not counterbalance the evolutionary effect of fishing. Our model also produced relative maturation sizes that are broadly consistent with empirical estimates for many fish species.

## Graphical abstract

Fishing is a strong evolutionary force, but its interplay with predation remains unclear. Using a community size‐spectrum model we show that fishing has strongest evolutionary effect on large predatory species, but minor impact on small species. Middle‐sized species are sandwiched between fishing and predation and their responses are highly variable.

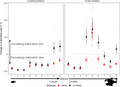

## 1 INTRODUCTION

The last century has been marked by a rapid decline in the health of many ecosystems due to exploitation, invasive species, climate change, pollution, and eutrophication (Halpern et al., 2008; Smith, 2003). These drivers represent strong selective pressures, and rapid evolutionary responses have been documented in many organisms and ecosystems (Darimont et al., 2009; Palumbi, 2001; Sullivan et al., 2017). In marine ecosystems, one of the major ecological and evolutionary forces is fishing (Audzijonyte et al., 2016; Fugère & Hendry, 2018; Jorgensen et al., 2007). Fishing can alter body size structure, size‐specific mortality, optimal life histories, and lead to evolution toward earlier maturation, smaller adult body sizes, and altered behavior (Audzijonyte et al., 2013; Conover & Munch, 2002; Therkildsen et al., 2013). Evolution of maturation and body size in response to size‐selective fishing has been demonstrated in experimental studies (Conover & Munch, 2002; Uusi‐Heikkilä et al., 2015) and single‐species models (Enberg et al., 2009; de Roos et al., 2006). However, outcomes of experimental studies cannot be easily extrapolated to real ecosystems, because it is unclear how ecological and evolutionary feedbacks through species interactions might modify selection pressures imposed by fishing (Kuparinen & Merilä, 2007).

Life‐history theory makes it clear that increased adult mortality will select for earlier maturation (Charnov et al., 2013). Although wild fish stocks around the world have been observed to follow a trend toward earlier maturation and smaller maximum body size (Audzijonyte et al., 2013, 2016; Olsen et al., 2005), debate remains as to whether this can be explained by fishing‐induced evolution (FIE) (van Rijn et al., 2017). Multiple drivers can affect maturation and body size and similar changes are also observed and expected in response to increased water temperatures (Audzijonyte et al., 2016; Baudron et al., 2014; Blanchard et al., 2005). The combined effect of these pressures on trait evolution is not straightforward to predict. For example, high predation can also drive evolution to earlier maturation in wild populations (Reznick et al., 1997, 2008) and predation on small individuals may override evolutionary selection from low fishing intensity, and even drive an increase in maturation size (Edeline et al., 2007). Furthermore, increased and decreased maturation size were both observed in a single predator‐single prey model, that included competition and cannibalism, when increased mortality was applied to small individuals; this is because change in maturation size proved to be dependent on how mortality changed with body size (Claessen et al., 2002; Gårdmark et al., 2003). As fishing pressure increased in many intensively harvested areas, predation mortality has declined substantially, due to large changes in the biomasses and size structure of top predators (Fisher et al., 2010), leading to large effects on the abundance of smaller species (e.g., Shackell et al., 2010). These studies suggest that a universal decrease in maturation size in response to fishing may be unlikely in complex multi‐species ecosystems, where multiple species are fished and interact through predation and competition. Understanding and predicting FIE in a multi‐species context therefore requires better representation of the potential interactions between fishing and other ecological selection forces.

The debate on the universality of FIE has important implications for precautionary fisheries management. If evolutionary responses to fishing are unpredictable and varied, it is unrealistic to expect its inclusion in forecasts of stock productivity. It would also mean that the widespread trends toward earlier maturation in many harvested stocks could be caused by factors other than, or in addition to, fishing (e.g., climate change) (see Audzijonyte et al., 2016; van Rijn et al., 2017). On the other hand, if under most conditions fishing does select for earlier maturation at smaller body size in multi‐species systems, fisheries managers should be encouraged to account for such trends in their management plans. To address the role of species interactions and eco‐evolutionary feedbacks on the evolution of fish maturation size under fishing, we used a multi‐species size spectrum model with temporal adaptive evolution of maturation size. The need for this kind of model is well recognized (e.g., Fraser, 2013), yet most marine ecosystem and multi‐species models do not include selection‐driven (as opposed to random) evolutionary changes (Belgrano & Fowler, 2013).

Individual body size is widely accepted as one of the most important functional traits, especially in marine ecosystems, and size spectrum food‐web models have been successfully applied to study changes in individual body size distributions of communities and ecosystems (Blanchard et al., 2017). Size spectrum models can resolve the detailed demography of species by characterizing maturation and asymptotic sizes, as well as enabling given sizes of particular species to interact with other sizes and species through predation (including cannibalism) and competition (Hartvig & Andersen, 2013).

The inclusion of maturation size in size spectrum models makes them particularly useful for addressing questions related to adaptive maturation responses to fishing. Indeed, these kinds of models have previously been coupled with adaptive dynamics models to explore the long‐term effects of different selective forces on maturation sizes. For example, interference competition, in combination with predation but without fishing, has been shown to influence the distribution and diversity of maturation sizes at equilibrium in a modeled community size spectrum (Zhang et al., 2015). Fisheries‐induced evolution has also been studied using a similar modeling framework. Law and Plank (2018) used a two‐species size‐spectrum model to explore the effects of different size‐structured harvesting strategies on maturation size changes, emphasizing the importance of including both intra‐ and interspecific predation. They also suggested that to usefully inform contemporary fisheries management, closer examination of the intricacies of multi‐species systems at shorter time scales would be warranted (Law & Plank, 2018). The adaptive dynamics approach used in these and other models (Dieckmann & Law, 1996; Gårdmark et al., 2003) assumes a separation of ecological and evolutionary timescales, with fast ecological dynamics influencing selection acting on species and size classes and the introduction of new species occurring at equilibrium. However, evidence of rapid evolution and eco‐evolutionary feedbacks is well recognized and ubiquitous (Beckerman et al., 2016; Ellner, 2013; Matthews et al., 2016), meaning that ecology and evolution happen on the same timescales.

Inspired by the above adaptive dynamics studies, we develop a model that allows us to investigate the consequences of traits adapting and changing through time, but with the introduction of new phenotypes occurring at the same timescale as the ecological processes of feeding, growth, mortality, and reproduction. We have extended the physiologically structured multi‐species size spectrum modeling approach to explore temporal eco‐evolutionary dynamics of maturation size and its response to fishing. With this model, we tackle the central questions regarding FIE, namely whether and how ecological interactions (e.g., intra‐ and interspecific predation) affect fisheries‐induced selection pressures on maturation size (Carlson et al., 2007; Edeline et al., 2007; Kuparinen & Merilä, 2007). We ask three main questions. Does, in accordance with single‐species predictions, FIE lead to universally declining maturation size? How does the interaction of fishing versus predation (and cannibalism) pressure affect the emergent maturation size for species of different asymptotic sizes and hence different trophic roles? What is the minimum fishing intensity necessary to trigger FIE responses in maturation size?

We expect that strong size‐selective fishing will select for earlier maturation size. However, we expect that predation (which for our purposes includes cannibalism) will also lead to changes in maturation size, but that the direction of these changes is harder to predict because predatory interactions are more complex than the pressures arising from size‐selective fisheries. We explore whether predation can counterbalance the evolutionary pressure from fishing in species at different trophic levels and assess at what level of intensity fishing becomes the overpowering selective force.

## 2 METHODS

**FIGURE 1:**
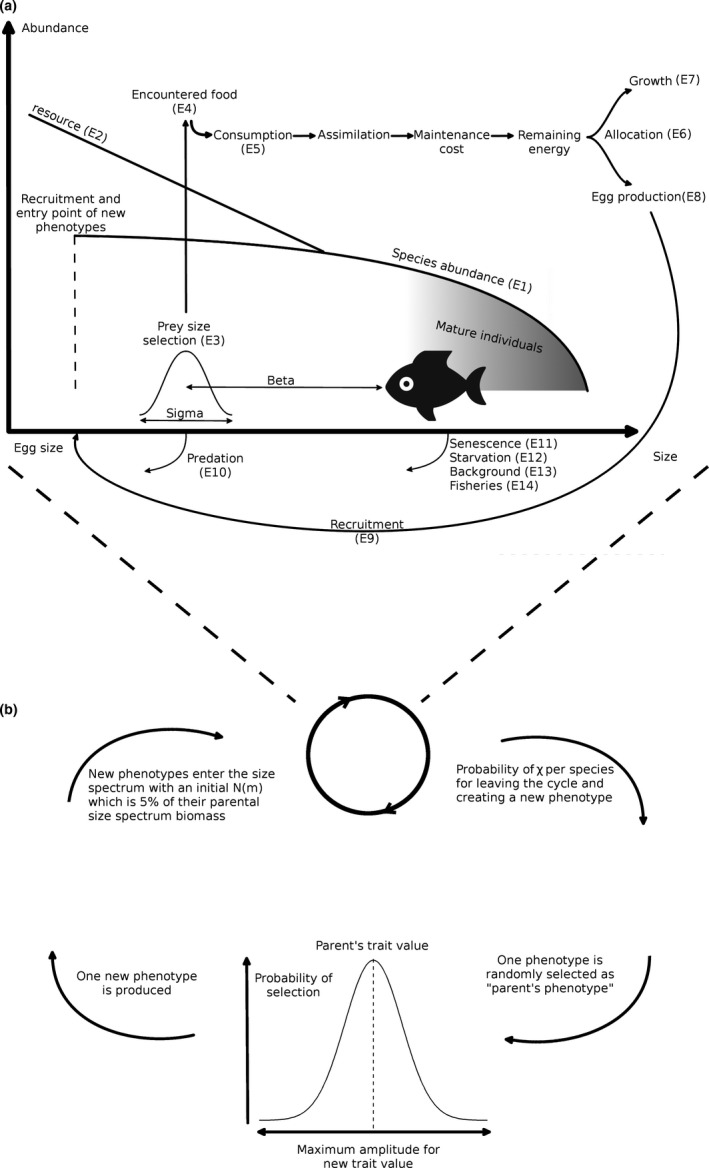
Schematic representation of key model components. (a) shows the energy pathways occurring in the model through the different equations found in Table 2. E1, E2, and E9 govern the abundance of the different components. E3 to E5 are used for energy intake which is then divided between growth E7 and reproduction (E8) depending on the maturation state (E6). Mortalities are described by Equations E10–E14. (b) shows the evolutionary processes built around the ecological model

In this study, we explore the eco‐evolutionary feedbacks between fishing, community dynamics, and dynamic temporal changes (evolution) in maturation size. To model community dynamics, we used a modified version of the trait‐based size spectrum model (Andersen & Pedersen, 2010; Hartvig et al., 2011) implemented in the R package “mizer” (Scott et al., 2014; which also provides further documentation). Our modifications to “mizer” include the introduction of eco‐evolutionary dynamics (the code is available on https://github.com/baldrech/MizerEvo). The modeling approach has two components: (a) an ecological component, which defines intra‐ and interspecific interactions that act as selective forces influencing survival and community dynamics (i.e., as in the basic “mizer” package), and (b) an evolutionary component that generates random new trait values (i.e., maturation size), which are introduced in the community at each time step. A conceptual model illustration is shown in Figure 1 and below we describe the key components of the model with equations and parameters provided in Tables 1, 2, 3 (for further details on the assumptions in the “mizer” package see https://sizespectrum.org/mizer/).

**TABLE 1:** Initial maturation, asymptotic size, and *R*_max_ of the species

Species	Maturation size (g)	Asymptotic size (g)	*R*_max_
1	2.5	10	0.49208
2	8	32	0.19854
3	25	100	0.08011
4	79	316	0.03232
5	250	1,000	0.01304
6	790	3,162	0.00526
7	2,500	10,000	0.00212
8	7,905	31,622	0.00085
9	25,000	100,000	0.00034

**TABLE 2:** Model equations for each species' ecological dynamics

Equation	Description	
Species population dynamics	$\frac{\partial N\left(m\right)}{\partial t}+\frac{\partial }{\partial m}\left(g\left(m\right)N\left(m\right)\right)=‐\mu \left(m\right)N\left(m\right)$	E1
Background resource dynamics	$\frac{\partial N_{R}\left(m,t\right)}{\partial t}=r_{0}m^{p‐1}\left[\kappa_{R}m^{‐\lambda }‐N_{R}\left(m,t\right)\right]‐\mu_{p}\left(m\right)N_{R}\left(m,t\right)$	E2
Prey size selection by size *m* predator	$\phi \left(m,m_{p}\right)=\exp\left[\frac{‐{\left(\ln\left(m/\left(\beta m_{p}\right)\right)\right)}^{2}}{2\sigma^{2}}\right]$	E3
Encountered food by size *m* predator across all sizes (*mp*) of species *j* prey	$E\left(m\right)=\gamma m^{q}\int \left(N_{R}\left(m_{p}\right)+\sum_{j}\theta_{j}N\left(m_{p}\right)\right)\phi \left(m,m_{p}\right)m_{p}dm_{p}$	E4
Feeding level	$f\left(m\right)=\frac{E(m)}{E\left(m\right)+hm^{n}}$	E5
Energy allocation toward reproduction	$\psi \left(m\right)={\left[1+{\left(\frac{m}{m^{*}}\right)}^{‐u}\right]}^{‐1}{\left(\frac{m}{M}\right)}^{1‐n}$	E6
Somatic growth	$g\left(m\right)=\left(\alpha f\left(m\right)hm^{n}‐k_{s}m^{p}\right)\left(1‐\psi \left(m\right)\right)$	E7
Reproduction	$R_{p}=\frac{\varepsilon }{2m_{0}}\int N(m)\left(\alpha f\left(m\right)hm^{n}‐k_{s}m^{p}\right)\psi \left(m\right)dm$	E8
Recruitment	$R=R_{\max}\frac{R_{p}}{R_{p}+R_{\max}}$	E9
Predation mortality on size *mp* prey inflicted by all sizes (*m*) of species *j* predators	$\mu_{p}\left(m_{p}\right)=\sum_{j}\int \phi \left(m,m_{p}\right)\left(1‐f_{i}\left(m\right)\right)\gamma_{j}m^{q}\theta_{j}N_{j}\left(m\right)dm$	E10
Senescence mortality	$\mu_{\mathrm{se}}=\left\{\begin{array}{cc} se_{\min}+\left(se_{\max}‐se_{\min}\right)e^{‐\frac{M}{m}} & \text{if}\;m>m*\\ 0, & \text{otherwise} \end{array}\right)$	E11
Starvation mortality	$\mu_{\mathrm{st}}=\left\{\begin{array}{cc} st_{r}m\left(k_{s}m^{p}‐\alpha f\left(m\right)hm^{n}\right), & \text{if}\;k_{s}m^{p}>\alpha f\left(m\right)hm^{n}\\ 0, & \text{otherwise} \end{array}\right)$	E12
Background mortality	$\mu_{b}=\mu_{0}M^{n‐1}$	E13
Fishing mortality	$\mu_{f}=\left\{\begin{array}{cc} 0.8, & \text{if}\;m\geq m*\\ 0, & \text{otherwise} \end{array}\right)$	E14

Note

Subscripts for each species are not included in the equations below for readability. These dynamics also hold for each phenotype (nested within each species) once they have entered the size spectrum. Equation numbers reference the processes illustrated in Figure 1 and descriptions in main text. *N*(*m*) is the density at size driven by: *g*(*m*) which is the feeding dependent growth rate at size and *μ*(*m*) which is the mortality at size, the latter is comprised of several mortality terms below. All parameter values and definitions are provided in Table 3 or the main text.

**TABLE 3:** Parameters table

Symbol	Value	Units	Parameter
Individual growth			
*M*	Variable	g	Asymptotic size
*η*	0.25	–	Ratio between *M* and m*
m*	*ηM*	g	Maturation size
*f*_0_	0.5	–	Initial feeding level
*γ*	538	g^−q^ m^3^ year^−1^	Factor for search volume
*α*	0.6	–	Assimilation efficiency
*h*	85	g^1−^ *^n^* year^−1^	Maximum food intake
*n*	0.75	–	Exponent for max. food intake
*p*	0.75	–	Exponent for standard metabolism
*ks*	4	–	Factor for standard metabolism
*β*	100	–	Preferred predator‐prey mass ratio
*σ*	1	–	Width of the feeding kernel
*q*	0.8	–	Exponent of search volume
Reproduction			
*m*_0_	0.1	mg	Offspring mass
*ε*	1	–	Efficiency of offspring production
*u*	7	–	Width of maturation transition
Mortality			
*ξ*	0.1	–	Fraction of energy reserves
*μ*_0_	2	g^1‐^ *^n^* year^−1^	Background mortality
*se*_max_	1	g^1‐^ *^n^* year^−1^	Upper limit for senescence mortality
*se*_min_	0.1	g^1‐^ *^n^* year^−1^	Lower limit for senescence mortality
*st_r_*	0.1	year^−1^	Starvation mortality cost
*θ*	Variable	–	Interaction matrix species‐specific value
Resource spectrum			
*κ*	0.05	g^λ−1^ m^−3^	Magnitude of resource spectrum
*λ*	2 − *n* + *q*	–	Slope of resource spectrum
*r*_0_	4	g^1‐p^ year^−1^	Regeneration rate of resources
*m*_cut_	1	g	Upper limit of resources spectrum
Evolution			
*χ*	0.001	–	Phenotype's introduction probability
Ω	10^−30^	Individual/m^3^	Extinction threshold
	5%	–	Fraction of initial phenotype's abundance
	+−20%	–	Magnitude of trait evolution

Note

M is noted "variable" as it is species specific (values in Table 1). *η* starts at 0.25 for all species but will evolve during the simulations. Parameters values are taken from Hartvig et al. (2011). The value from *γ* was calculated from: $\gamma =h\frac{f_{0}}{\sqrt{\left(2\pi \right)\sigma \beta^{\lambda ‐2}e^{\left({\left(\lambda ‐2\right)}^{2}\sigma^{2}/2\right)}\kappa \left(1‐f_{0}\right)}}$ (Hartvig et al., 2011). Initial values for the abundance density *N*(*m*) of each species at *t* = 0 were based on the equilibrium equation: $N=\frac{\kappa }{1000}M^{\left(2n‐q‐2+0.35\right)}m^{\left(‐n‐0.35\right)}$ provided in Andersen and Beyer (2006).

### 2.1 Ecological component

Size spectrum models are physiologically structured models that track the density of individuals at size through time (Andersen, 2019). The trait‐based approach means that the model has several “species” or size spectra, which differ only in their asymptotic and maturation size (maturation size is assumed to be a fraction of asymptotic size). This modeling approach has the desired combination of model simplicity (only one set of physiological parameters required), but still allows for multiple species with variable maturation sizes.

Growth, maturation, and reproduction are all food dependent, and driven by the process of size‐dependent predation, and the model therefore includes emergent intra‐ and interspecific competition. This means that the evolution in response to fishing and predation is studied against a backdrop of underlying ecological competition. Feeding, growth, mortality, and reproduction occur at every time step. The flux of individuals between size bins depends on growth (inflow from smaller size bin, outflow to larger size bin) and mortality. The population dynamics of each species are then obtained by solving the conservation equation (von Foerster, 1959; McKendrick, 1926):

$$\frac{\partial N(m)}{\partial t}+\frac{\partial }{\partial m}\left(g\left(m\right)N\left(m\right)\right)=‐\mu \left(m\right)N\left(m\right).$$

where *m* is the species mass and individual growth *g*(*m*) and mortality *μ*(*m*) are determined by predation on/from other individuals, and a background resource spectrum modeled using a semi‐chemostat growth assumption (Table 2, E2).

#### 2.1.1 Food consumption

In this model, all individuals are simultaneously predators and prey, where no distinction is made between interspecific predation and cannibalism. The available food comes from all of the fish species and a background resource size spectra, which here is assumed to extend from 10^−10^ to 1 g (bacteria to zooplankton) (Table 3, with the same regeneration rates as in Hartvig et al. (2011). All species begin life at the same size (0.001 g) and compete for food in the resource size spectrum. As they grow larger the extent to which they feed on themselves and each other is dictated by a species interaction matrix and size‐based feeding kernel. A species by species interaction matrix scales the proportion of available biomass of each prey species to each predator species, with the diagonal setting the intensity of cannibalism. Here, we used two contrasting symmetric interaction matrices with all values set to either 0.5 or 0, depending on whether predatory interactions were included or not (see below). We chose 0.5 to define the predatory interactions, assuming that prey biomass is never completely available to predators at any given time, due to spatial or temporal separation and predator avoidance behavior. When the interaction matrix is set to 0 all sizes and species compete for food in the resource size spectrum. Encountered food is the product of the volumetric search rate that scales with body size and the availability of food within the size spectrum (Hartvig et al., 2011).

Whether or not encountered prey are eaten is determined by a size‐dependent feeding kernel with the preferred predator:prey mass ratio *β* and width of the feeding kernel *σ* (Table 3), and is described by a log‐normal selection model:

$$\phi \left(m_{p},m\right)=\exp\left[\frac{‐{\left(\ln\left(m/\left(\beta m_{p}\right)\right)\right)}^{2}}{2\sigma^{2}}\right].$$

where *m_p_* is prey mass and *m* the predator mass.

Once the available size range has been determined, the realized food consumption is modeled through a standard Holling type II functional response, determined by the search rate and maximum intake rate, resulting in the emergent feeding (satiation) level (Table 2, E4–E5).

#### 2.1.2 Growth

The consumed food is assimilated with an efficiency *α* and the resultant energy is divided between metabolism and growth (Table 2, E7), with the latter further divided between somatic growth and reproduction (Table 2, E6) depending on the maturation status. Resource allocation between growth and reproduction follows a logistic curve, where half of the growth resources are allocated to reproduction at maturation size, making fecundity scale with body size. We have modified E6 from Hartvig and Andersen (2013) and the “mizer” default equation by changing the scaling parameter (*u*, Table 3) of energy allocation to reproduction from 10 to 7 to allow for a more prolonged period between minimum and maximum investment in reproduction.

#### 2.1.3 Reproduction

New recruits enter the smallest size class at every time step, that is, the model assumes continuous reproduction (Table 2, E8 and Table 3). Recruitment is determined using the Beverton‐Holt type stock–recruitment relationship (Andersen & Pedersen, 2010), defined by Equation E9 (Table 2) and the maximum flux recruitment parameter *R*_max_ (Table 1) (see below for further details on recruitment in the evolutionary model). An upper limit on the recruitment flux (*R*_max_) is used to impose additional density dependence otherwise not captured by the processes in the ecological model, but that are recognized to be important in marine fish populations (which also leads to an emergent stock–recruitment relationship; e.g., Andersen et al., 2016).

#### 2.1.4 Mortality

In addition to the emergent predation mortality, other sources of mortality include senescence mortality (Table 2, introducing survival cost of reproduction, for example, Kuparinen et al. (2012), E11), starvation mortality (Table 2, E12), a constant background mortality where larger species are assumed to have lower background mortality (Hartvig et al., 2011; Table 2, E13), and fishing mortality (Table 2, E14).

### 2.2 Evolutionary component

In this study, we explore evolutionary changes in a single trait ‐ maturation size. This was modeled through the *η* parameter, which defines the fraction of the theoretical asymptotic size at which 50% of an individual's net energy is allocated to reproduction (Table 3). We chose to modify *η* rather than asymptotic size (as in Zhang et al., 2015) to ensure that dynamic change in *η* only affects the resource allocation and the emergent growth, but not the background mortality which depends on theoretical asymptotic size (Table 2, E13). Changes in *η* through time were modeled similarly to the unstructured eco‐evolutionary food‐web model of Allhoff et al. (2015) by introducing new size spectra (“phenotypes”) characterized by new trait combinations. In contrast to Allhoff et al. (2015), however, who ran their simulation to equilibrium before adding new phenotypes (mutations), we allowed for a possibility of new phenotypes to appear at each time step, assuming a constant influx of new mutations. Our approach assumes no interbreeding among phenotypes (new genetic variation is only generated via the mutation process), because each phenotype produces offspring identical to itself and no intermediate trait values among phenotypes emerge. This approach is also similar to that used in adaptive dynamics (Dieckmann & Law, 1996). As the simulations ran, each species generally had 10–50 phenotypes (Figure S10, showing the number of phenotypes per species through time), turning over their abundances through time in response to the selection forces at play. Our approach approximates temporal dynamics of *η* in response to selection by tracking each phenotype through time and computing the changing mean and variance of *η* for each species.

These new phenotypes were generated by randomly selecting an already existing phenotype (i.e., set of parameter values) within a species to represent a “parent.” At each time step, there is an equal probability that each species will generate a new phenotype. The new phenotype is a copy of its parent except for the maturation size for which values are randomly drawn from a normal distribution ranging from −20% to 20% of the parent's trait value (Figure 1). The initial abundance of the new phenotype was assumed to be 5% of the parent's biomass, which is subtracted from the parent's biomass. This means that phenotypes of less abundant parents have low initial abundance and lower chance to become established in a population, to ensure that the realized rate of evolution depends on the population size. Following their entrance into the ecosystem at egg size, the phenotypes compete for food and are predated upon, and hence change in abundance. The extinction threshold was set at Ω = 10^−30^ ind.m^−3^ (Hartvig et al., 2011) and all phenotypes below this density were removed. The probability of new phenotype appearance *χ* was set to 0.001 per time step and the initial *η* values were assumed to be 0.25 for all species (Hartvig & Andersen, 2013). This combination of parameters produced an expected evolutionary rate similar to that observed in populations with high fisheries intensity (Audzijonyte et al., 2013, see Section 4). To ensure robustness of these parameters for our findings, we explored the sensitivity of different values of *χ* and initial *η* along with other key parameters (Appendix 1).

#### 2.2.1 Balancing extinction and coexistence through food limitation

We focus on the eco‐evolutionary interactions between predation and fishing against the backdrop of food limited conditions that includes inter‐ and intraspecific competition for resources. Food limitation is needed to enable some competition, extinction of less fit phenotypes, and temporal change in maturation size. The application of a maximum recruitment *R*_max_ assumes strong density dependence early in life; lower *R*_max_ leads to lower recruitment and reduces competition for a given resource density, while high *R*_max_ leads to competitive exclusion by one or a few species (Andersen et al., 2016). Because we are focussed on modeling evolutionary changes against the backdrop of food limitation, the initial *R*_max_ values were set for each species assuming the default values provided in the trait‐based model of “mizer” (see also Andersen, 2019) and predation/resource parameters were calibrated (*σ*, *κ,* and *r*_0_, Table 3) to ensure a balance of coexistence and food limitation (feeding levels between 0.12 and 0.7) (Table 1).

The phenotypes behave like separate species in that their size spectra are tracked independently and they compete with each other. However, all phenotypes in one species are affected by the same *R*_max_. This means that during reproduction, all offspring are pooled within each species, one *R*_max_ applied to all of them to calculate the new offspring numbers, and these are then distributed among phenotypes in proportion to their spawn output (i.e., a phenotype with a high spawn output will have more recruits with its traits than a phenotype with a low spawn output). Thus, abundant phenotypes are not disproportionally affected by *R*_max_, which would be the case if it was applied to each phenotype separately.

#### 2.2.2 Fitness calculations

As phenotypes are constantly being introduced and becoming extinct, the resulting fitness landscape and eco‐evolutionary dynamics are ever‐changing. We calculate fitness landscapes at several time intervals to explore the selection pressures on phenotypes through time.

We track the cohort survival and fecundity through time for 50 years (*t*_max_), using a modified version of *R*0 (lifetime reproductive output) as a proxy for fitness:

$$\text{fitness}=\frac{\int_{t_{0}}^{t_{\max}}R_{\max,i}\frac{R_{p,i}}{R_{p,i}+R_{\max,i}}dt}{N_{p,i}\left(m_{1},t_{0}\right)}$$

where *R_p,i_* is the energy allocated to reproduction (E8) by phenotype *p* of species *i*, *R*_max,_ *_i_* is the maximum recruitment value for species *i* and *N_p,i_* (*m*_1_, *t*_0_) is the initial numbers of phenotype *p* and species *i* in the cohort of interest. This measure is similar to “eggs per recruit” (Andersen, 2019). We used this fitness calculation to construct snapshots of fitness gradients (in relation to maturation size) for all species' phenotypes across all simulations to assess whether modeled directional changes are consistent with these gradients. Because different species have different *R*_max_ values, the fitness cannot be quantitatively compared across species, but they are comparable across phenotypes within a species where the same *R*_max_ value applies.

### 2.3 Simulation design

To assess how predation affects evolution of species' maturation size under fishing, we conducted simulations using four different model scenarios—with and without fishing and with and without predatory interactions (interaction matrix set to 0.5 or 0, respectively). In all simulations, we used a model composed of 9 species with asymptotic sizes equally spread on a logarithmic scale between 10 to 10^5^ g. The initial abundance of each species was determined based on the equilibrium conditions (Andersen & Beyer, 2006), which uses feeding and carrying capacity parameters to estimate biomass at equilibrium. When predatory interactions are disabled all species only feed on the background resource spectrum, but they still compete for food.

Fishing was imposed through a knife‐edge selectivity function, where all fish at or above the selected size were subjected to an instantaneous fishing mortality rate. For simplicity and to minimize the number of alternative fishing scenarios, the selected size for all species is set at 0.25 of asymptotic size (i.e., at the initial maturation size). For the main set of scenarios, we applied the instantaneous fishing mortality *F* = 0.8 per year, as this was high enough to trigger ecological and evolutionary responses, but sufficiently low to avoid extinctions, and represents fishing pressure historically applied to many fish stocks (RAM Legacy Stock Assessment Database, 2020). To assess the sensitivity of model outcomes to fishing intensity, we also explored the results with fishing mortality ranging from 0.1 to 1.0 per year.

Since the eco‐evolutionary dynamics and fitness values change throughout the simulations as new phenotypes appear, the model is not necessarily expected to reach equilibrium conditions. To account for stochasticity of the eco‐evolutionary dynamics, each scenario (parameter combination) was repeated 10 times. To test whether 10 stochastic realizations were enough to capture trends, we also ran all predation‐enabled scenarios with 50 replicates but found no substantial difference in trait changes and variances (See Figures S7 and S8 in Appendix 1).

The simulations were run for 3,000 years without fishing to allow the ecosystem to build up multiple phenotypes per species, establish evolutionary trends in the absence of fishing and for the influence of initial conditions to dissipate. After 3,000 years, the full simulation state of each stochastic run was saved and used to initiate two additional 3,000 years of simulations, with and without fishing imposed. The effects of fishing on the evolutionary dynamics were assessed by comparing dynamics and final states in simulations with and without fishing.

The sensitivity of the model outcomes to the parameter values was assessed across a range of: fishing mortalities (0.1–1); the initial trait value *η* (Figure S1); the standard deviation between parent and new trait (Figure S2); new phenotype appearance rate *χ* (Figure S3); the initial phenotype abundance (Figure S4); width of the feeding kernel *σ* (Figure S5); and preferred predator‐prey mass ratio *β* (Figure S6).

### 2.4 Analyses of simulation outputs

We assessed the modeled communities by exploring phenotypes' abundance history (i.e., phenotype biomass time series including when they appear and go extinct) and evolutionary trends of each species' average relative maturation size *η*. This was calculated as the abundance weighted mean trait value across all phenotypes for each species in each simulation through time. Since the simulations were conducted as a factorial design (predation x fisheries) we tested whether final values of *η* (after 6,000 years) depended on the interaction of Species*Predation*Fishing using a three‐way Model 1 (fixed effects) ANOVA, where Predation (0 or 1), Fishing (0 or 1) indicate presence or absence of predation and Species is the species number, with *n* = 10 replicate simulations. To ensure among‐group homogeneity of variance in residuals, we log‐transformed *η* (after transformation the homogeneity was achieved).

All three terms and their interactions were evaluated using *F*‐tests and model comparisons were carried out using delta AIC tests. While we report p‐values, the purpose of these models was to evaluate and interpret the nature of the interactions in the model. Statistics were done using R version 4.0.0 (R Core Team, 2020), the 'effects' (v4.1‐4; Fox et al., 2019) and the “emmeans” (v1.4.7; Lenth et al., 2020) packages. The lower and upper 95% confidence intervals for all model predicted effects were calculated using the allEffects function in the R package “effects.”

To assess whether the final values of maturation size (*η*) were compatible with empirical observations, we compared our results with empirical estimates for wild fish stocks (Conover & Munch, 2002; Goodwin et al., 2006; Jennings et al., 1998; Olsson & Gislason, 2016; Reznick et al., 1997; Rijnsdorp et al., 1992; Ulloa et al., 2011).

## 3 RESULTS

### 3.1 Maturation size trajectories

**FIGURE 2:**
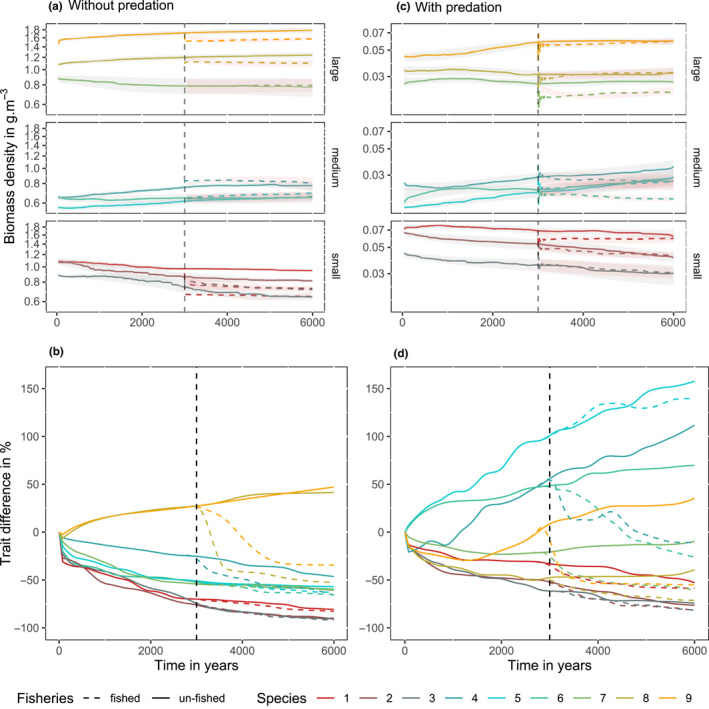
Biomass and trait variation averaged and smoothed throughout all simulations in scenarios without predation (a, b) and with predation (c, d). (a, c) Is the change in species' biomass, the gray shading indicates the standard deviation across simulations. (a, c) Is divided in 3 panels (small, medium, large being the species asymptotic size) for more clarity. The vertical line shows the introduction of fishing and from this line, the dashed lines are the biomass when fished, with the red shading showing its standard deviation. (b, d) Is the proportional change in weighted mean maturation size for each species relative to its initial value averaged across all stochastic realizations. The vertical line shows the time where fishing is introduced, and the dashed line shows simulations with fisheries

During the initial 3,000 years of simulations without fishing, the presence of inter‐ and intraspecific predation had large effects on the evolution of maturation sizes (Figure 2). In simulations without predation, the relative size at maturation decreased substantially (40%–90%) in all but the largest two species (Figure 2b, before the dashed line). In contrast, when predation was enabled the evolution of maturation size diverged across the three size groups, where the smallest three species evolved toward smaller maturation (decrease by 45%–60%), the middle‐sizes species toward larger maturation size (increase by 50%–150%), and in the largest three species the trait evolved slightly in either direction (Figure 2d, before the dashed line). After the initial 3,000 years, the trend of evolution in scenarios without fishing generally continued in the same direction at a slower rate or stabilized for the remaining 3,000 years (difference between the dashed line and solid line in Figure 2b,d).

**FIGURE 3:**
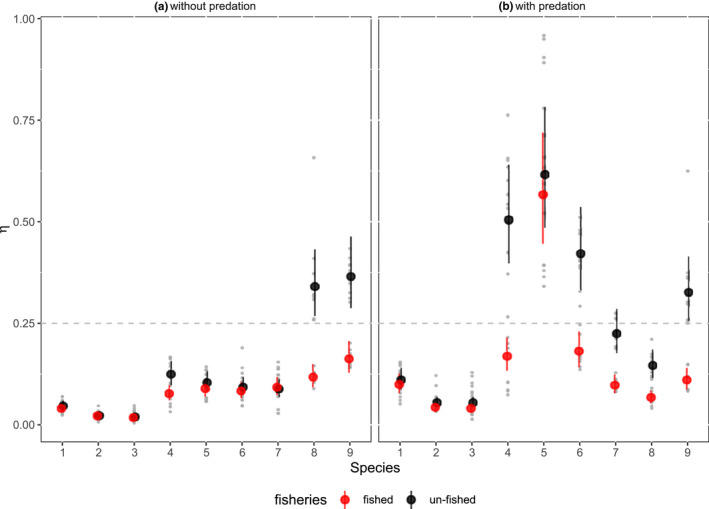
Final maturation size (relative to each species' asymptotic size) (*η*) at the end of the simulation (6,000 years). Each dot shows the predicted means and 95% confidence intervals from the 3‐way ANOVA model for each species, predation and fisheries combination. Data points show weighted average *η* for each species (across 10–50 phenotypes, see Figure S10 for the number of phenotypes per species) where each light gray point is a replicated simulation (10 simulations for each combination of fisheries and predation scenarios). The dashed line indicates the initial relative maturation size value (*η*)

After the introduction of fishing at year 3,000, the biomass of many species quickly reached a lower state (Figure 2a–c dashed lines), but then recovered in some species as they evolved to adapt to new mortality regimes. In all species and scenarios, fishing led to either decreasing or static maturation size, the latter mostly occurring in cases where maturation size has already evolved to be less than 50% the starting value (except species 5 with predation, Figure 2d). For the entire ecosystem, the decline in maturation size due to fishing was stronger when predatory interactions were enabled compared to simulations without predation (strong decrease in maturation size for 5 out of 9 versus 2 out of 9 species in the community, Figure 2b–d), suggesting that fishing had a large effect on the ecosystem with predation. Our statistical analyses of the model simulations showed that the effect of fishing (red dots in Figure 3) reduced maturation size in medium and large species. All terms of the model were significant (Table S2), and there was a significant three‐way interaction among species, predation, and fishing (ANOVA: *F* = 2.6, *df* = 8.324, *p* = .009). The differences between maturation size between fished and unfished simulations clearly depended on the species and whether or not predation was enabled. Significantly lower maturation sizes in the presence of fishing were found for species 4, 8, and 9 without predation (pairwise comparison tests, *p* < .005) and 4, 6, 7, 8, and 9 with predation (pairwise comparison tests, *p* < .001). Across stochastic realizations of simulations, variation in the final maturation size values was generally small for all species when predation was disabled, especially when variation is considered as a proportion of the *η* (i.e., species with larger *η* have larger absolute variations in Figure 3, but the proportional variation is similar). With predation enabled, variation was small in the three smallest species, and very large in the two medium‐sized species (species 4 and 5) (Figure 3). Sensitivity analyses showed that the observed impacts of fishing were qualitatively similar across a range of tested parameter values (see Appendix 1).

### 3.2 Maturation size changes in relation to fitness gradients

**FIGURE 4:**
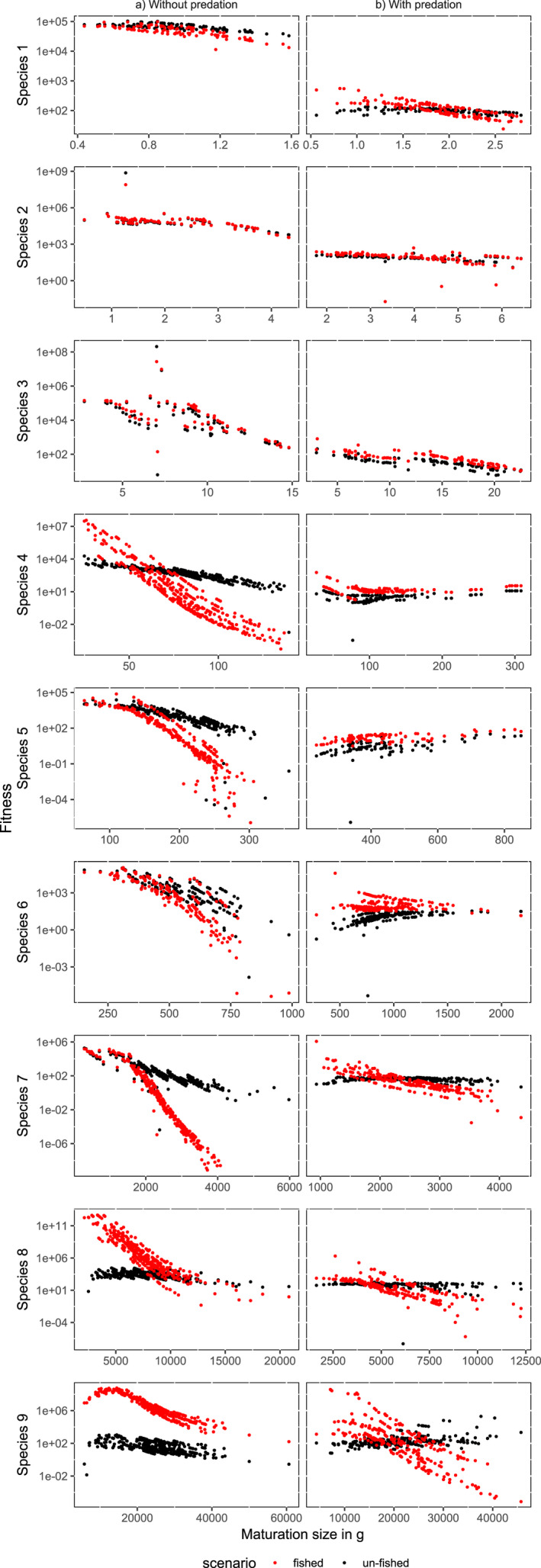
Species' fitness across simulations. Each dot shows fitness of a phenotype with different maturation size in scenarios with (red) and without (black) fishing across simulations. The figure shows phenotypes in a cohort starting at year 3,001 in simulations without (a) and with (b) predation, following the introduction of fisheries. Fitness landscapes in year 5,500 were generally similar and shown in Figure S9

The species‐specific maturation sizes responses to fishing and predation can be in part understood by examining their fitness gradients at a given time (Figure 4 shows these at the onset of fishing in year 3,000, and Figure S9 shows them close to the end of the simulation period). For the smallest species, neither predation nor fishing had much effect on the fitness gradient, where phenotypes with smaller maturation sizes had slightly higher fitness values, explaining the observed constant and slow evolution toward smaller maturation size. Introduction of fishing generally did not alter the shape of the fitness gradients for these species (red dots compared to the black dots, Figure 4, top three rows). In contrast, the medium‐sized species' response to fishing strongly depended on whether or not predation was enabled. When predation was disabled, smaller maturation sizes of medium‐sized species had higher fitness (Figure 4 center three rows, left panels), explaining their evolution toward smaller body sizes (Figure 3 no predation panel) whereas the opposite was generally true when predation was enabled (except in species 4, where the fitness gradient was not monotonic and either the smallest or largest maturation sizes had the highest fitness). Introduction of fisheries only steepened the fitness gradients in scenarios without predation (both in year 3,000 and year 5,500, Figure S9), making smaller maturation sizes even better adapted while for scenarios with predation the effect of fishing on fitness landscapes was less defined, with large variation across phenotypes (the difference in fitness gradients was more clear for species 6). This response can explain the large variation in the evolutionary trends across stochastic simulations for species 4 and 5 (Figure 2d), suggesting that eco‐evolutionary dynamics may be less predictable for the medium‐sized species because of the interplay of selection pressures from fishing and predation. Finally, the effect of fishing was especially clear on the fitness gradients of the largest species, where smaller maturation sizes always had higher fitness under fishing, and phenotypes with large maturation sizes were largely absent after 2,500 years of fishing (Figure 4 and Figure S9).

### 3.3 Impact of increasing fisheries effort

**FIGURE 5:**
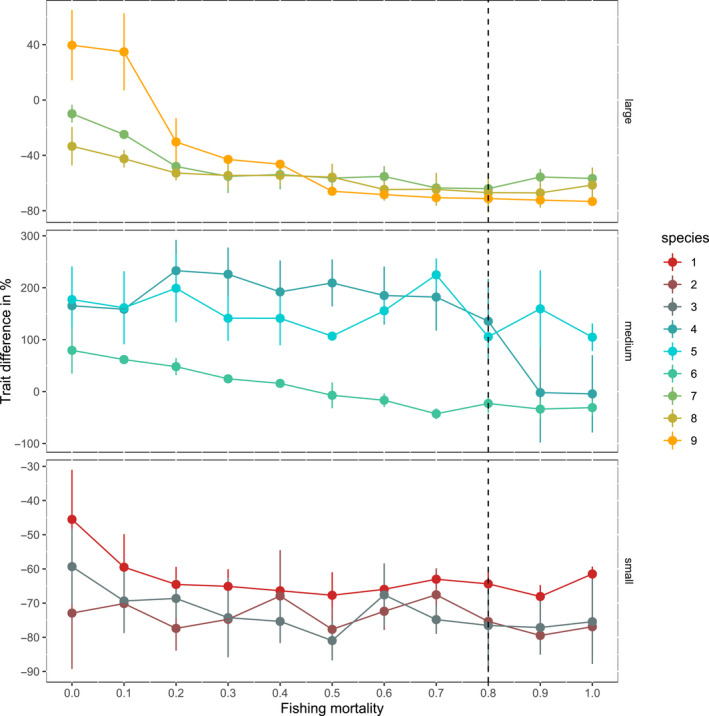
The effect of the instantaneous fishing mortality rate (per year) on changes in maturation size in scenarios with predation. Each line shows the biomass weighted average trait value at the end of the simulation for each species. Error bars show the standard deviation across simulations. The vertical dashed line shows the default parameter values used in the study

We explored how changes in the intensity of fishing affected trait evolution, by repeating simulations with predation for 10 values of instantaneous fishing mortality rate—from 0 to 1 per year. For the largest four species (species 6–9), the only level of fishing mortality that did not lead to strong decrease in maturation size was 0.1 per year. Increasing fishing mortality to 0.2 per year caused maturation size to decline. Indeed maturation size stayed relatively stable at this new level for all mortality values above 0.4 per year (0.7 per year for species 6) (Figure 5). For the three smallest species changes in fishing mortality did not have much effect, because their maturation size was close to the smallest possible given the physiological trade‐offs assumed in the model (i.e., reproductive output is size dependent, so at least some growth is needed for reproduction to occur) and the computational constraints of the model where the maturation size was reached in just a few time steps. Finally, two of the medium‐sized species (species 4 and 5) had large variance in their maturation sizes. For species 5, where fishing did not significantly reduce the maturation size, increasing fishing effort also did not have a clear effect. For species 4, clear effects of fishing on maturation size were seen only at high fishing mortalities (*F* ≥ 0.8 per year).

### 3.4 Comparison with empirical patterns

**FIGURE 6:**
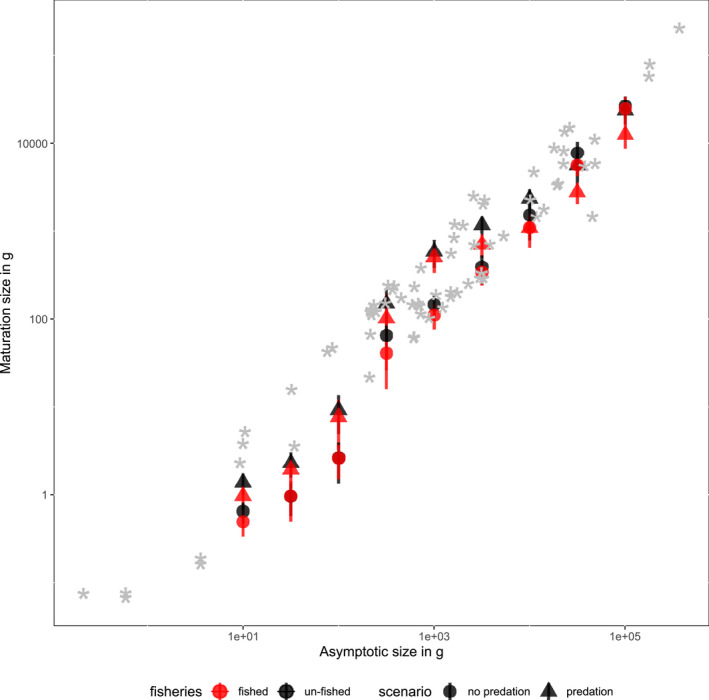
Modeled and empirical relationship between maturation size and asymptotic size. Modeled estimates of maturation size are from the end of the simulation period (year 6,000) with (red) and without (black) fishing and with (triangle) and without (circle) predation. Error bars show the standard deviation across simulations. Silver asterisks are values obtained from empirical studies of fish species for comparison (see Table S3 for data and references)

We found that despite divergent evolution of maturation sizes the emergent relationship between the maturation size and maximum body size (at year 6,000) generally fell within the range of empirical relationship observed for marine fish across a range of body sizes (Figure 6). The overall slope of this relationship remained consistent across simulations with and without fishing.

## 4 DISCUSSION

The eco‐evolutionary model presented here aims to tackle the interplay between predatory interactions and size‐dependent fishing on the evolution of maturation size. The model uses simple rules of size‐dependent predation, body size scaling of physiological processes, and introduction of random trait variation. There are four key findings of this study. First, we show that without any constraints on the direction and limits of maturation size, substantial changes in the evolutionary trends in maturation size emerge, but these changes are broadly consistent with empirically observed patterns. Second, we show that both predation and fishing are strong selective forces, but their effects interact and differ across species of different asymptotic sizes. For the small species, regardless of predation and fishing, species evolved toward smaller maturation sizes. In contrast, predation completely reversed selection gradients in the medium‐sized species, while fishing generally reversed selection and trait evolution in medium and large species. Third, in agreement with single‐species predictions, fisheries generally led to smaller maturation sizes for all but the smallest three species, and its effects were stronger in ecosystems with predation (and cannibalism) enabled. Fourth, for the largest species even low fishing mortality (0.1 per year) was enough to drive evolutionary change toward smaller maturation size, as predation mortality was considerably lower than fisheries mortality at the largest sizes.

### 4.1 Predation and emergent maturation size

The role of predation on optimal maturation size has been addressed in a range of models, including size spectrum approaches, generally suggesting that maturation sizes diverge to promote species coexistence (Allhoff et al., 2015; Hartvig & Andersen, 2013; de Roos et al., 2006; Zhang et al., 2015). However, these studies used an adaptive dynamics approach and assessed optimal fitness or invasion success of new traits in equilibrium conditions, separating ecological and evolutionary timescales. While the findings of these studies are important, they did not include continuous ecological‐evolutionary feedbacks known to shape natural systems (Govaert et al., 2019). Evolutionary feedbacks of species in a multi‐species system have previously been suggested to be equally important driver of community response as harvesting (Wood et al., 2018). This is consistent with our findings, which demonstrated that in a number of species the maturation size trajectories varied substantially through time and across stochastic realizations, even when biomass was relatively stable through time. This suggests that the timing and ecological conditions present at the time phenotypes emerged can greatly affect their success, particularly within medium‐sized species.

Despite the variation across stochastic realizations and temporal dynamics in trait values, one of the most consistent evolutionary trends seen in our study is the decreasing maturation size of the smallest species in all scenarios, irrespective of predation or fishing. This is illustrated by similar fitness trends across the scenarios (i.e., smallest maturation sizes had higher fitness for small species in all four scenarios of predation and fishing) and could partly be explained by the food limitation at around 10 g (seen as a drop in feeding level, see Figure S11). By evolving toward smaller maturation size, species divert fewer resources to growth, can stay longer in size groups between 1 and 10 g, benefit from better feeding conditions, but are not yet exposed to high predation (see panel b in Figure S11). Thus, trends in maturation size strongly depend on the resource availability, which is consistent with Hartvig and Andersen (2013), where optimal maturation and asymptotic sizes in a single‐ or two‐species size spectrum models were entirely determined by the resource density. Moreover, Hartvig and Andersen (2013) showed that the system can exist in different stable states depending on whether feeding limitation occurred at an early juvenile stage or around the maturation size (see Claessen & de Roos, 2003, for a similar finding in an age‐structured model). This means that some combinations of size‐specific resource limitation and maturation size are unstable and will select for either smaller or larger maturation size.

The key difference between Hartvig and Andersen (2013) and our study design is that the former explored the evolution of asymptotic size, while always setting the maturation size to be a fixed proportion of the asymptotic size. Yet, asymptotic size also determined background mortality (which can be high in small species) and the final evolutionary drivers (competition, predation, or differences in background mortality) were hard to identify. The factorial design of our simulations aimed to tease apart some of these drivers. Also, by specifically allowing for evolution of the *η* parameter, we allow the maturation size to evolve independently of the asymptotic size without affecting the background mortality. Additionally, we introduced the survival cost of reproduction (small increase in mortality after maturation), which are ubiquitous in nature and have an effect on evolution of maturation size (Kuparinen et al., 2012). In our design, the asymptotic size becomes a more theoretical parameter setting the largest possible body size. In reality, as maturation size, growth rates, and mortality evolve and change through time, many species never reach their asymptotic sizes. Nevertheless, both our and Hartvig and Andersen (2013) study share the same general finding—food limitation at around the maturation size will act as a strong selective force (for small species in our study).

Another notable finding is the evolution of maturation size in medium‐sized species, where predation reverses the maturation size trends and leads to a rapid increase rather than decrease in maturation size seen in scenarios without predation. Together with large variation in maturation sizes in middle‐sized species, this suggests that alternative maturation strategies might exist for these species, all dependent on the dynamic size‐specific mortality from predation and fishing. Although we did not study alternative stable states, our findings are consistent with, for example, Gårdmark and Dieckmann (2006) showing that such alternative stable solutions do indeed exist. As in Gårdmark and Dieckmann (2006), an important trade‐off in our model is the divestment of resources from growth to reproduction at around the maturation size. Delayed maturation size means that more energy at smaller size is available for growth and individuals will move faster to larger size classes, where they can potentially escape predation. The advantage of earlier or delayed maturation will therefore critically depend on the size at which predation mortality is lowest and feeding levels are highest (see also Duplisea, 2005; Pope et al., 1994).

### 4.2 Fishing and emergent maturation size

The response of maturation size to fishing has been studied using a range of size and age‐structured single‐species models (Andersen et al., 2007; Enberg et al., 2009; de Roos et al., 2006). Generally, these studies show that increased mortality due to fishing selects for earlier or smaller maturation size. For example, for the Baltic Sea cod (*Gadus morhua*), the optimal maturation size was predicted (based on single‐species and sized structured deterministic model) to be at least 10 times smaller than currently observed (Andersen et al., 2007). Yet, selection in wild populations is a tug of war among predation, pathogenic, competition, sexual selection, and human pressures (Carlson et al., 2007; Darimont et al., 2007; Edeline et al., 2007). For fish, in particular, predation is a powerful force, imposing strong selection on size, especially early in life (Perez & Munch, 2010), but also in adult individuals (Olsen & Moland, 2011). The strength of predation (including cannibalism) can counteract or even reverse evolutionary effects of fishing, such as in Lake Windermere pike *Esox lucius* (Edeline et al., 2007). It is therefore unclear how often and for which species harvest induced selection might be strong enough to override selection from predation or competition (e.g., Edeline et al., 2007; Eikeset et al., 2016; Kuparinen & Merilä, 2007).

Our results suggest that if predation is strong in early life stages and delayed maturation can help to outgrow this window, evolutionary effects of fishing can be particularly strong, as in some middle‐sized species (species 4 and 6). However, due to this predation versus fishing “tug of war” evolutionary impacts of fishing are not manifested until fishing mortality becomes relatively high (in species 4). Nevertheless, in one middle‐sized species (species 5) fishing did not reduce maturation size, as selection from predation, and possibly slightly improved food availability at slightly larger sizes (Figure S11) outweight the selection from fishing. While this may look like good news, such species might be particularly vulnerable to long‐term exploitation, unable to improve their fitness by evolving toward earlier maturation.

For large‐bodied species, the effect of fishing followed our expectations. In agreement with observations that harvesting imposes very strong selective pressures (e.g., Wood et al., 2018), fishing completely reversed natural selection gradients (in more realistic simulations with predation enabled) and led to a rapid evolutionary response of maturation size. The actual rate of response in our simulations cannot really be compared to real‐world ecosystems, because generation time, levels of phenotypic diversity and the genetic inheritance mechanisms in the model, do not accurately represent those in real fish populations. Nevertheless, the fastest rates of change observed in our model are broadly compatible with rates of change in empirically observed fish stocks or those predicted in ecogenetic models with more accurate evolutionary mechanism. The fastest rates observed in our simulations occur after the introduction of fishing and are in the range of 50% in 300 years or 0.17% per year (Figure 2b–d). In intensively fished stocks (*F* values similar to our baseline simulations) observed rates of phenotypic change are 1% per year, but this rate is likely to include both evolutionary and plastic trends. The rate (1% per year) is about four times faster that evolutionary rates reported in various population and ecogenetic models (Audzijonyte, et al., 2013), which is also compatible with our findings. The important result is that the evolutionary response occurred even at the instantaneous fishing mortality of 0.1 per year, which is generally considered a low level of fishing mortality, below levels that are consistent with maximum sustainable yield (Blanchard et al., 2014, www.ices.dk). This finding is consistent with other evolutionary models demonstrating that even low levels of fishing will select for smaller maturation size (e.g., Andersen et al., 2007).

### 4.3 Model limitations and future work

Although this study and modeling exercise is seemingly complex, it is a substantial simplification of real marine ecosystems. The fishing scenarios explored target all species with similar size selectivity and intensity and were stable through time. The initial set of species was spaced evenly over size categories and had shared diet preferences and other physiological parameters. None of this is true in the real world. We used an initial maturation size value of a quarter of the asymptotic size (Andersen & Pedersen, 2010; Hartvig et al., 2011) but different and even multiple optima may exist for species of different sizes. The evolutionary mechanism itself is highly simplified and does not include trait recombination or covariances, and each phenotype only produces offspring identical to itself. This means that selection differentials from the model cannot be compared with empirical studies. Finally, even though we included survival cost of reproduction, the full set of reproductive costs may still be too small. This could explain very small evolved maturation sizes in species—if a threshold amount of energy is required to achieve maturation, it would set a limit on how small maturation size could be. Indeed, a model with energetic cost of reproduction included (Audzijonyte & Richards, 2018) predicted a more realistic and larger maturation size of intensively fished Baltic Sea cod than a model without such costs (Andersen et al., 2007). Yet, despite these simplifying assumptions, we found a general emergent pattern of a conserved ratio of maturation sizes and maximum sizes that was consistent with empirical values (Figure 6). Our observed evolutionary rates were also broadly compatible with those expected in wild stocks. The range of *η* values from our model fell within the empirical range, noting that those values were obtained from species' maturation weight and weight at infinity from empirical von Bertalanffy relationships. Future studies would be worthwhile to further assess whether the predicted changes and timescales involved would still hold under more realistic species composition, traits, diets, and more realistic fishing scenarios.

## 5 CONCLUSION

One of the key questions in our study was to assess whether predation‐driven selection could counteract or even reverse fishing‐induced evolution (FIE). In this size and trait‐based food‐web model, the answer is that this is generally not the case, although it depends on the size of the species considered and fishing intensity imposed. Our findings suggest that for the largest species, harvesting, even at low intensity, imposes very strong selection because they have low predation mortality at around and above their maturation size. In contrast, the smallest species may be mostly limited by food availability, and neither predation nor fishing affect their fitness landscapes substantially. Such species may be already maturing close to their physiological or ecological limit, especially if cost of reproduction is considered (e.g., Audzijonyte & Richards, 2018). This shows the importance of simultaneously considering bottom up processes (e.g., food availability) when looking at FIE, and highlights the benefits of physiologically structured multi‐species models where growth and reproduction are dependent on food availability. Finally, the most unpredictable eco‐evolutionary responses emerge in medium‐sized species, sandwiched between larger predators, and smaller competitors. For these species, selection pressures from fishing, predation, and competition fluctuate through time and here predation release may indeed occasionally balance the selection from fishing. These findings call for more empirical studies on the possible evolutionary trends in medium‐sized species, improved understanding of interactive forces of selection, and stronger precautionary measures to minimize FIE in large fish.

## AUTHOR CONTRIBUTION

**Romain Forestier:** Conceptualization (equal); Formal analysis (lead); Methodology (equal); Software (lead); Visualization (lead); Writing‐original draft (lead); Writing‐review & editing (lead). **Julia Blanchard:** Conceptualization (equal); Formal analysis (supporting); Funding acquisition (lead); Methodology (equal); Project administration (lead); Supervision (lead); Writing‐original draft (lead); Writing‐review & editing (lead). **Kirsty L. Nash:** Supervision (supporting); Writing‐review & editing (equal). **Beth Fulton:** Supervision (supporting); Writing‐review & editing (equal). **Craig Johnson:** Supervision (supporting); Writing‐review & editing (equal). **Asta Audzijonyte:** Conceptualization (equal); Formal analysis (supporting); Methodology (equal); Supervision (equal); Writing‐original draft (lead); Writing‐review & editing (lead).

## Supporting information

Fig S1

Fig S2

Fig S3

Fig S4

Fig S5

Fig S6

Fig S7

Fig S8

Fig S9

Fig S10

Fig S11

Tbl S1

Tbl S2

Tbl S3

## Data Availability

The data and model open source code for this paper are available on https://github.com/baldrech/MizerEvo.

## APPENDIX 1 Sensitivity analysis

The robustness of our model outcomes to parameter assumptions was explored in an extensive set of simulations. In these sensitivity analyses, we are mostly looking at the qualitative difference in results, that is, whether the direction of evolutionary change in maturation size (up or down from the initial value) is the same as in the baseline scenarios. We are less concerned about the absolute quantitative change. Changing the initial *η* (Figure S1) resulted in different final *η* values for some of the medium‐sized species and, to a lesser extent, the large species, but the range of values we explored led to qualitatively similar trait changes by the end of the simulation and did not affect our overall conclusions. Regardless of the initial values of *η*, by the end of the 3,000 years, they all declined in small‐bodied species, and mostly increased in the middle‐sized species.

We assessed whether our assumptions about key predation parameters—predator‐prey mass ratio (*β*) and width of predation kernel (*σ*)—affected the direction (increase or decrease) in maturation size change at the end of 6000‐year simulation period. We found that, although the magnitude of the change varied somewhat, for all species, the direction of evolutionary trends was generally the same as in the baseline scenario. The only exception was for the highly variable species 4 and especially 5, when *β* or *σ* values were very small (50 and 0.9, respectively) (Figures S5 and S6).

Next, we explored sensitivity of model output to four parameters determining the rate of evolution: the magnitude of trait change, the phenotype appearance rate (*χ*), and the phenotype abundance upon introduction. The rate at which new phenotypes were introduced (*χ*) affected the speed of evolution, and almost no evolution occurred when the rate was 10 to 100 times slower than in the main simulation set (Figure S3). Yet, a 50% increase in *χ* gave qualitatively similar responses to fishing. Similarly, the magnitude of change between new and parent phenotypes determined the rate of evolution, where large amplitude of changes led to faster evolution, yet the qualitative response to fishing remained the same (Figure S2). Similarly, changing the initial abundance of a new phenotype along a continuum from 0.01% to 10% of the parent's abundance led to very similar evolutionary responses to those reported in the main text.

Finally, increasing the number of replications per scenario from 10 to 50 (in the scenario with predation and fishing) did not strongly affect the observed variation and demonstrated that 10 replications were largely sufficient to capture the variation and trends in trait evolution; the size of standard deviation bars remains the same across different replication values (Figures S7 and S8).
