# Supplementary figures and images for: Interacting forces of predation and fishing affect species’ maturation size
Source: Ecol Evol. 2020 Dec 5;10(24):14033–51. doi: 10.1002/ece3.6995 (PMC7771143; doi:10.1002/ece3.6995)

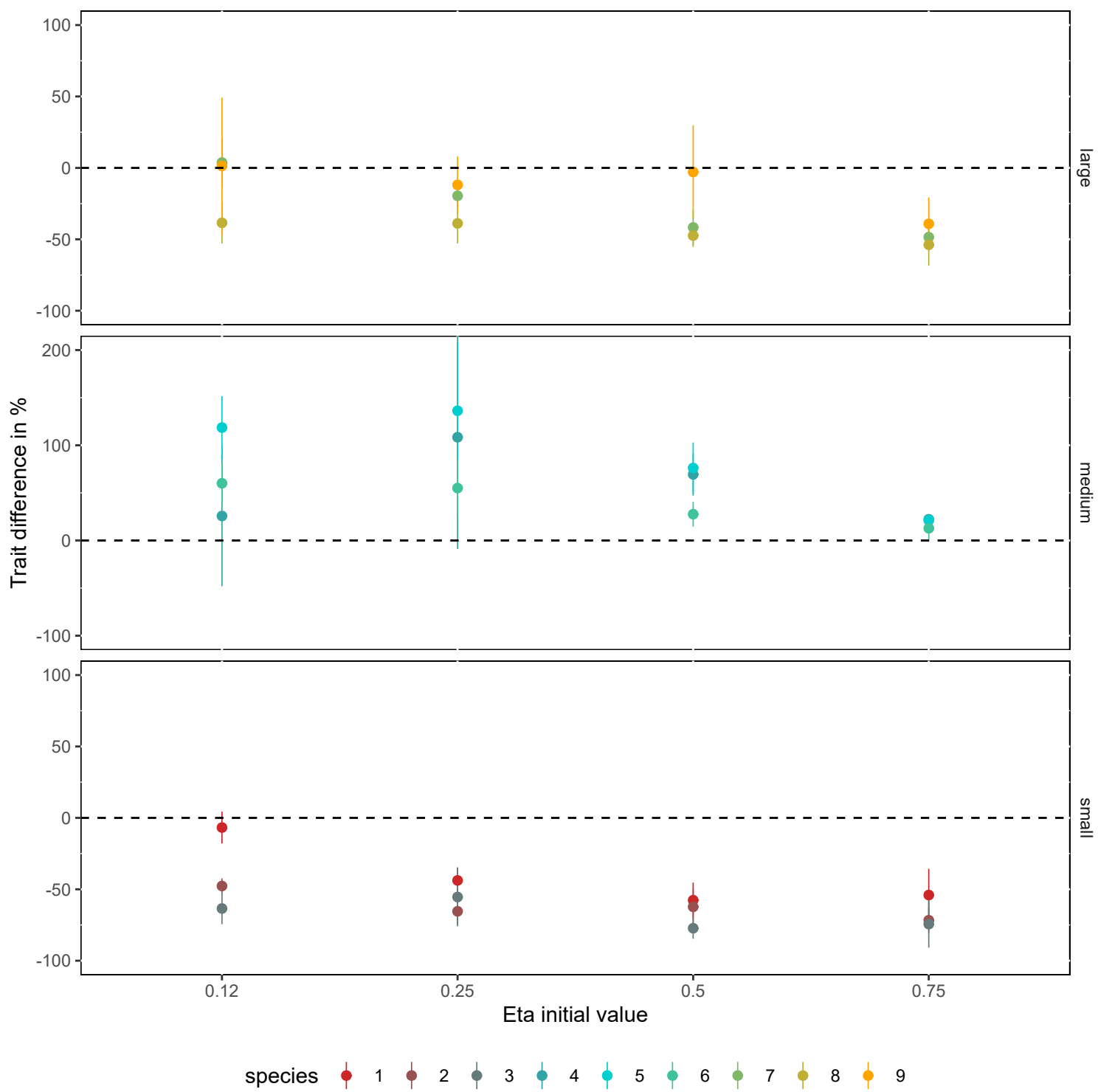

Supplement: Supplementary file 1 — Fig S1 [file ECE3-10-14033-s001.pdf]

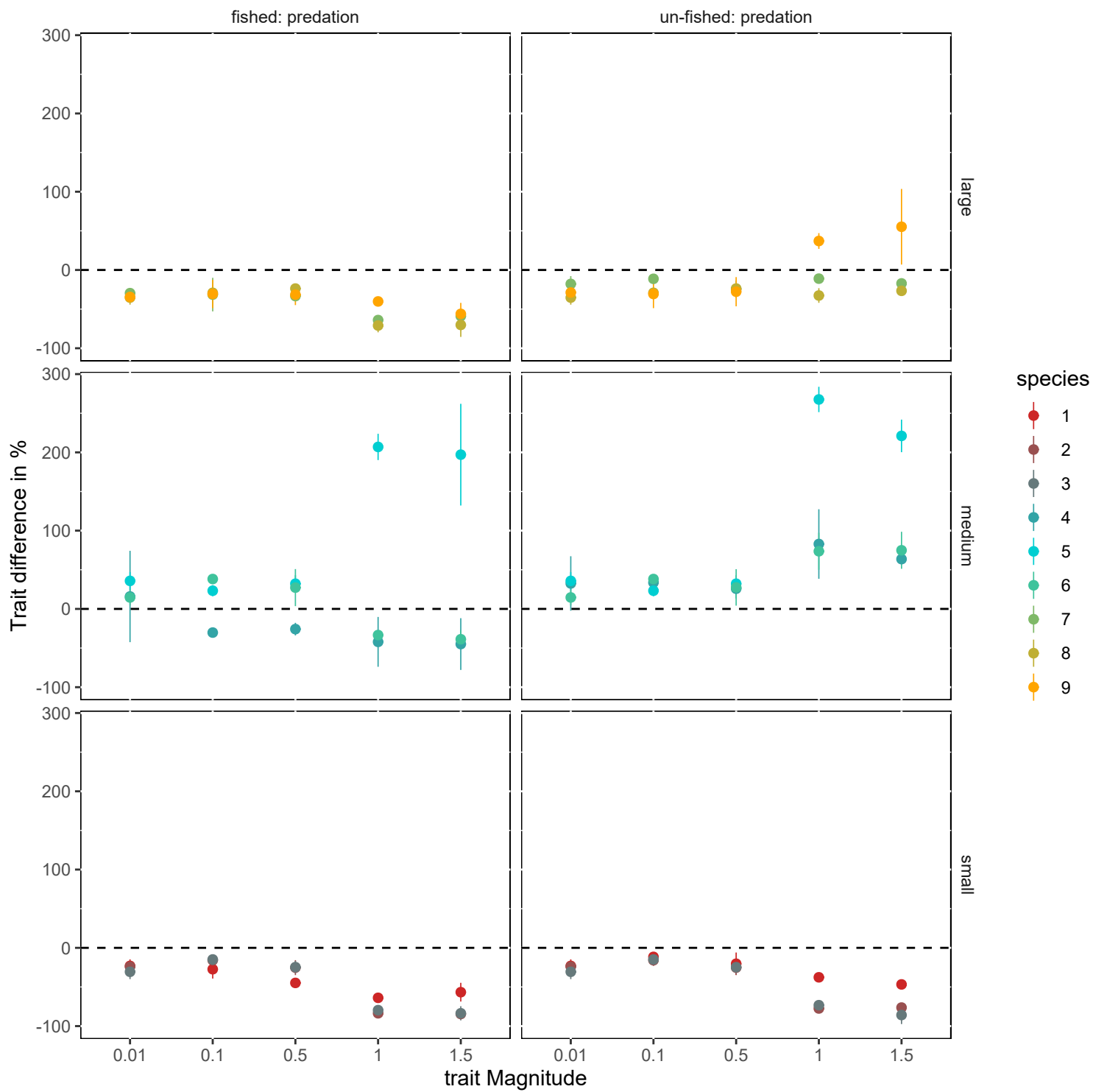

Supplement: Supplementary file 2 — Fig S2 [file ECE3-10-14033-s002.pdf]

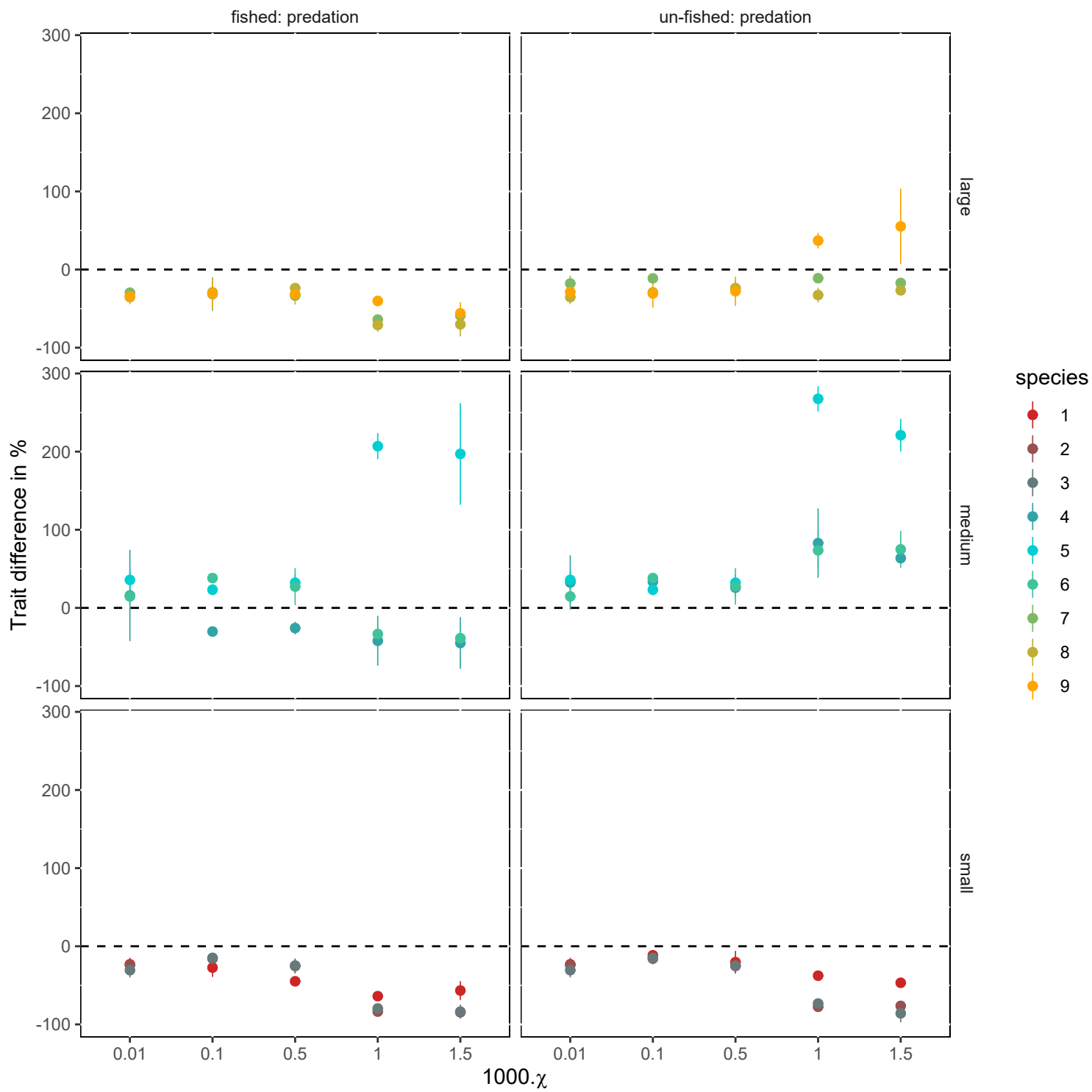

Supplement: Supplementary file 3 — Fig S3 [file ECE3-10-14033-s003.pdf]

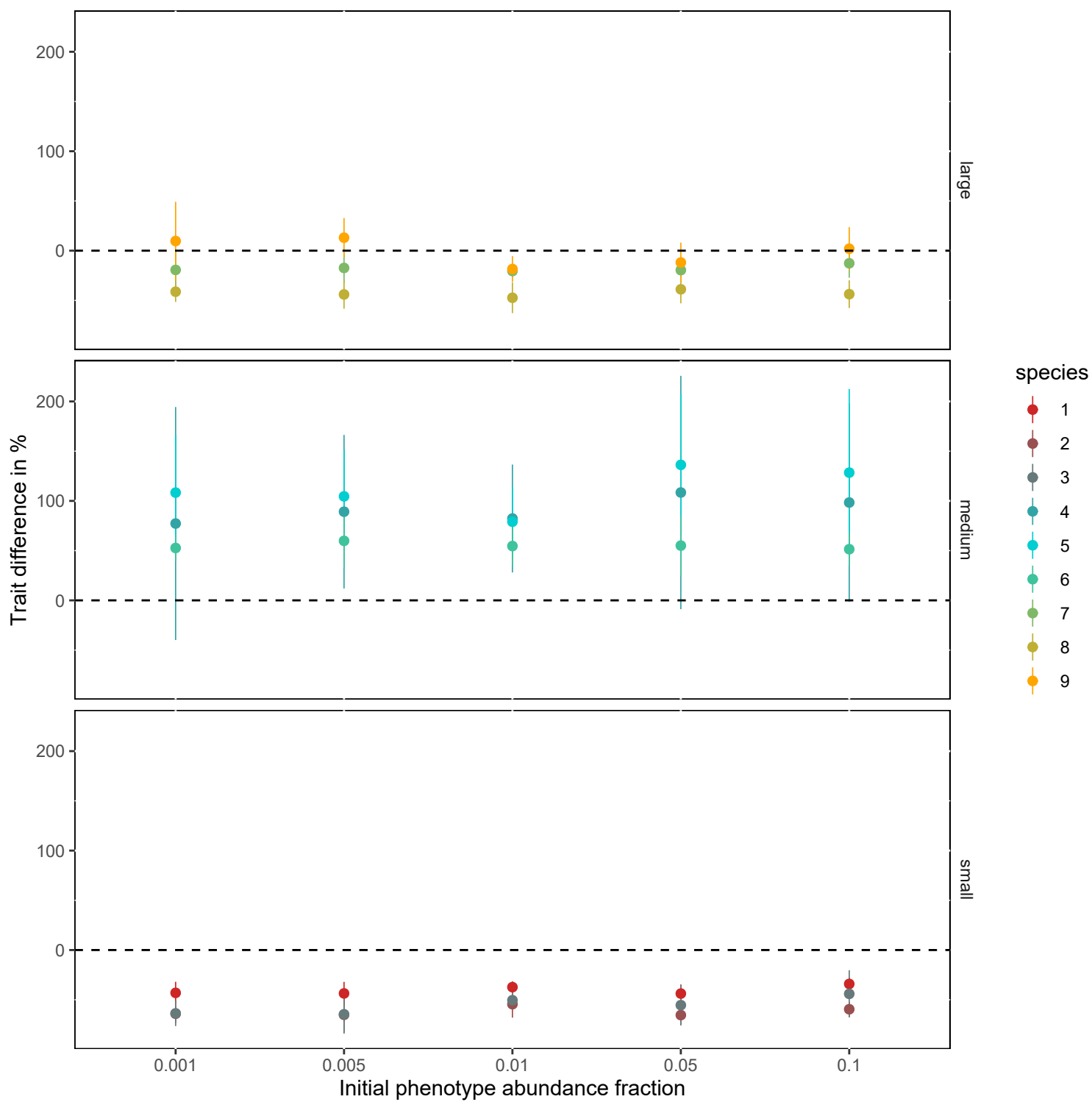

Supplement: Supplementary file 4 — Fig S4 [file ECE3-10-14033-s004.pdf]

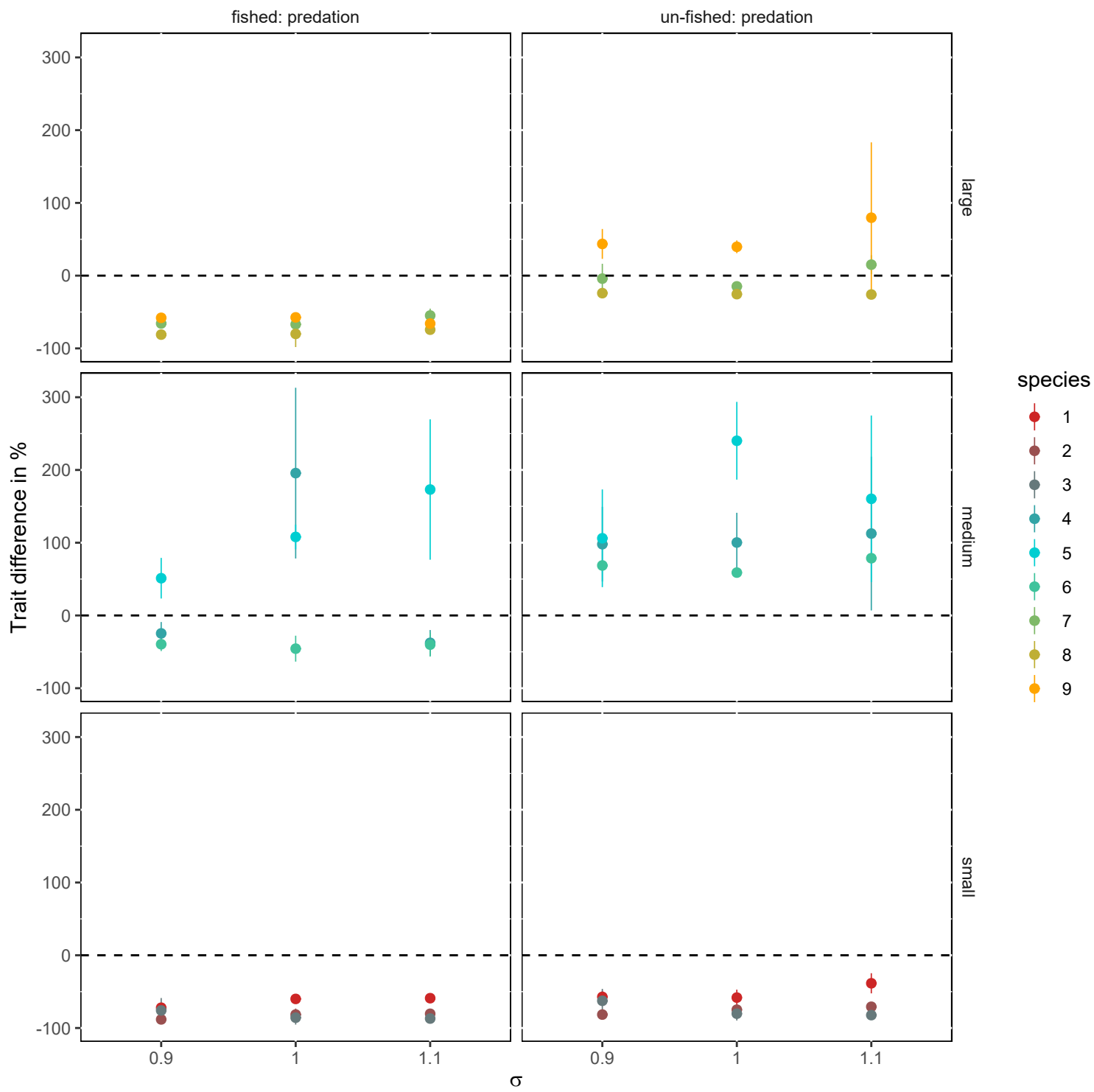

Supplement: Supplementary file 5 — Fig S5 [file ECE3-10-14033-s005.pdf]

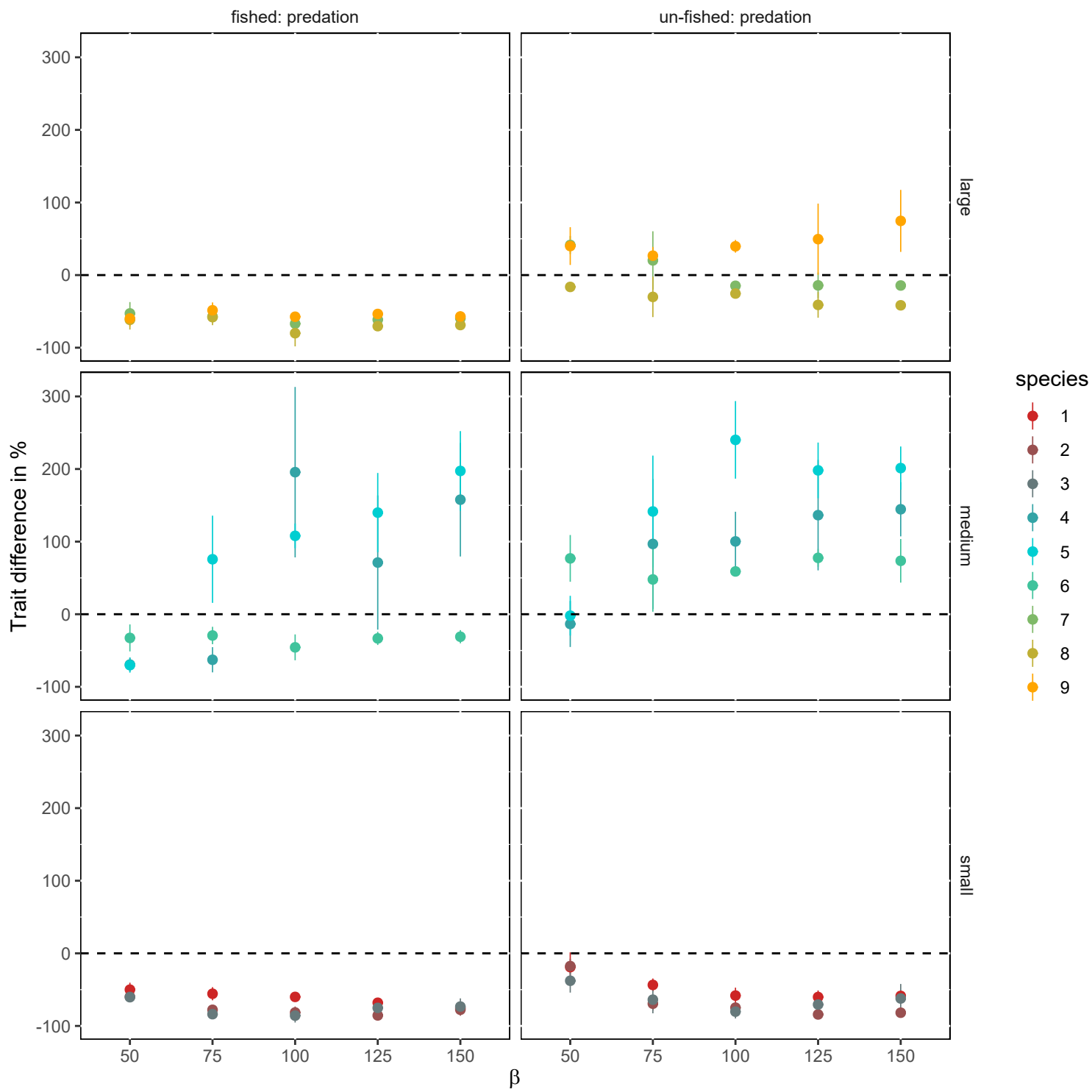

Supplement: Supplementary file 6 — Fig S6 [file ECE3-10-14033-s006.pdf]

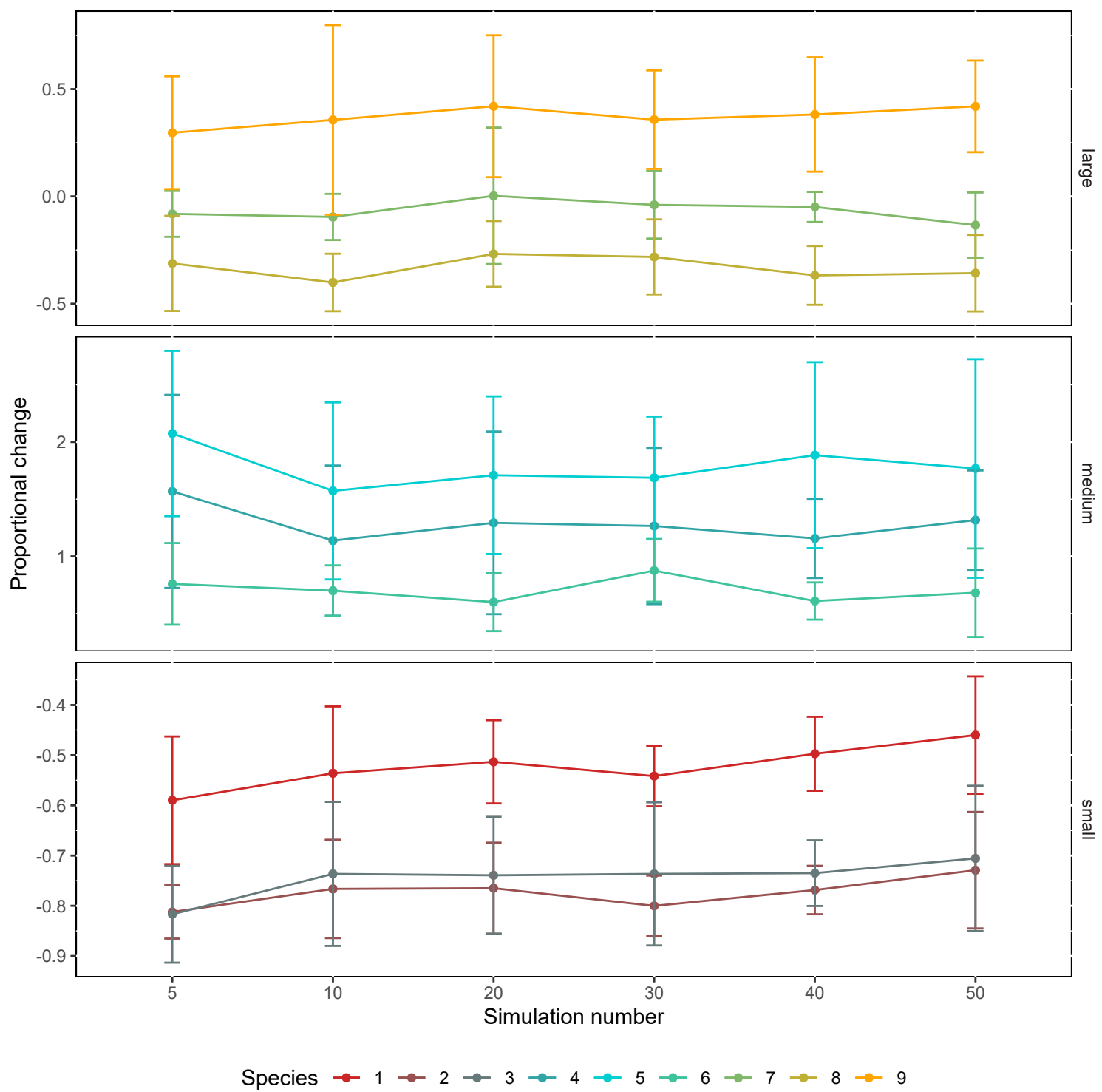

Supplement: Supplementary file 7 — Fig S7 [file ECE3-10-14033-s007.pdf]

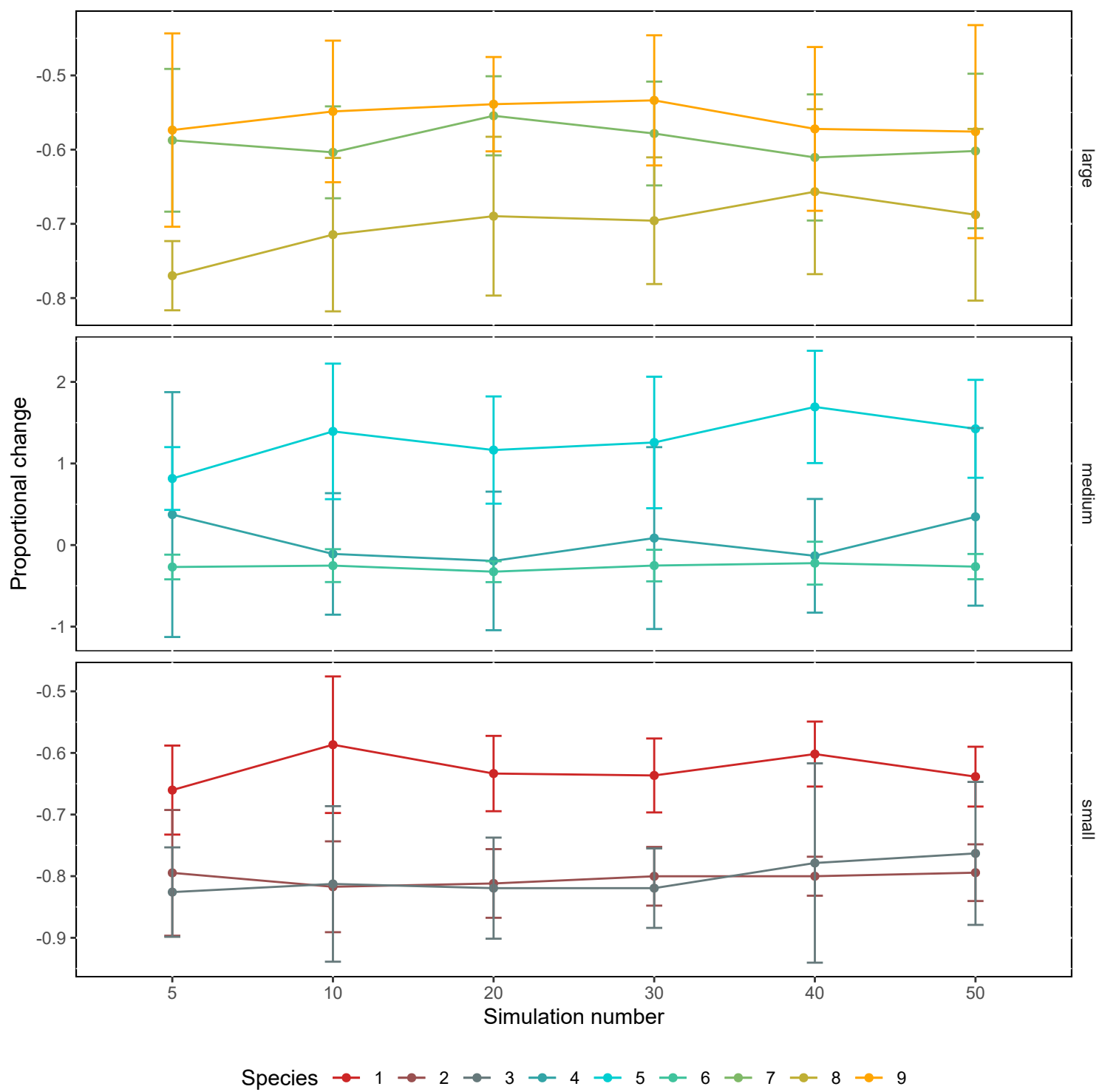

Supplement: Supplementary file 8 — Fig S8 [file ECE3-10-14033-s008.pdf]

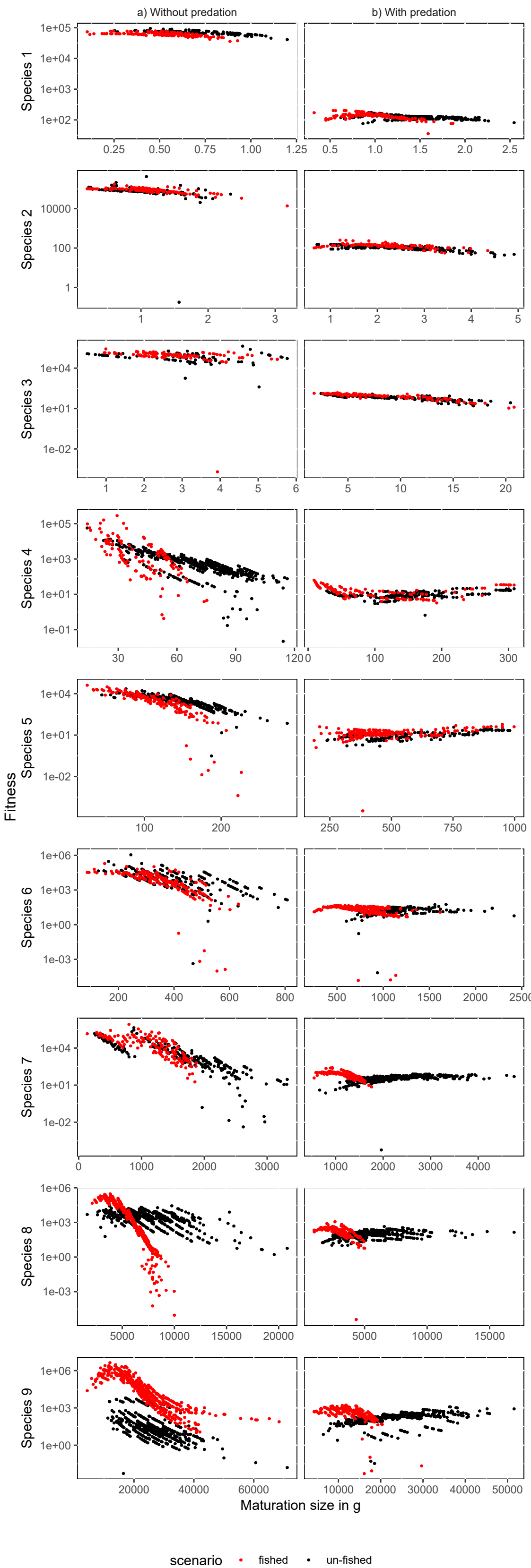

Supplement: Supplementary file 9 — Fig S9 [file ECE3-10-14033-s009.pdf]

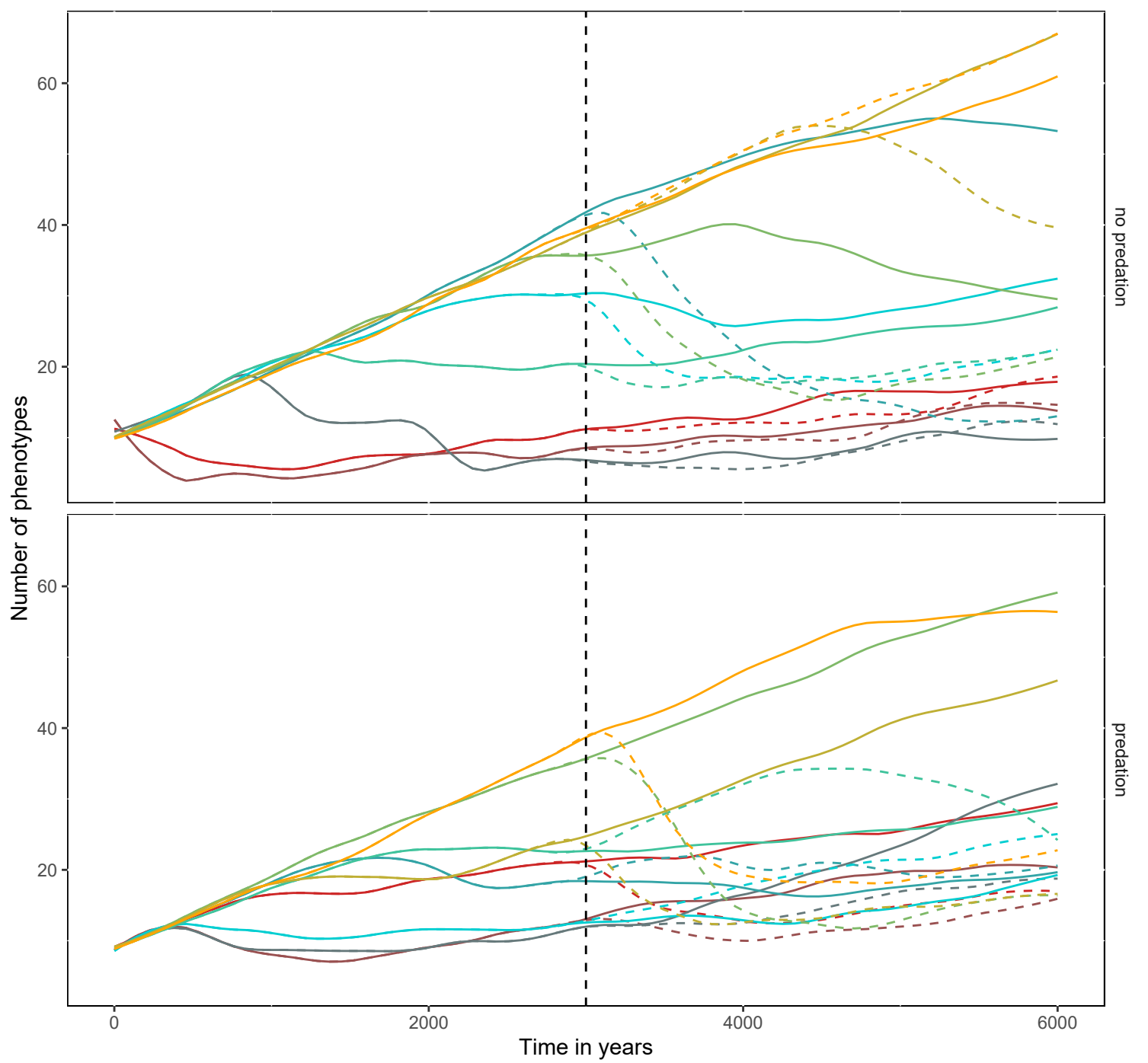

Fisheries    - - - fished    - - - un-fished

Species    - 1    - 2    - 3    - 4    - 5    - 6    - 7    - 8    - 9

Supplement: Supplementary file 10 — Fig S10 [file ECE3-10-14033-s010.pdf]

a)

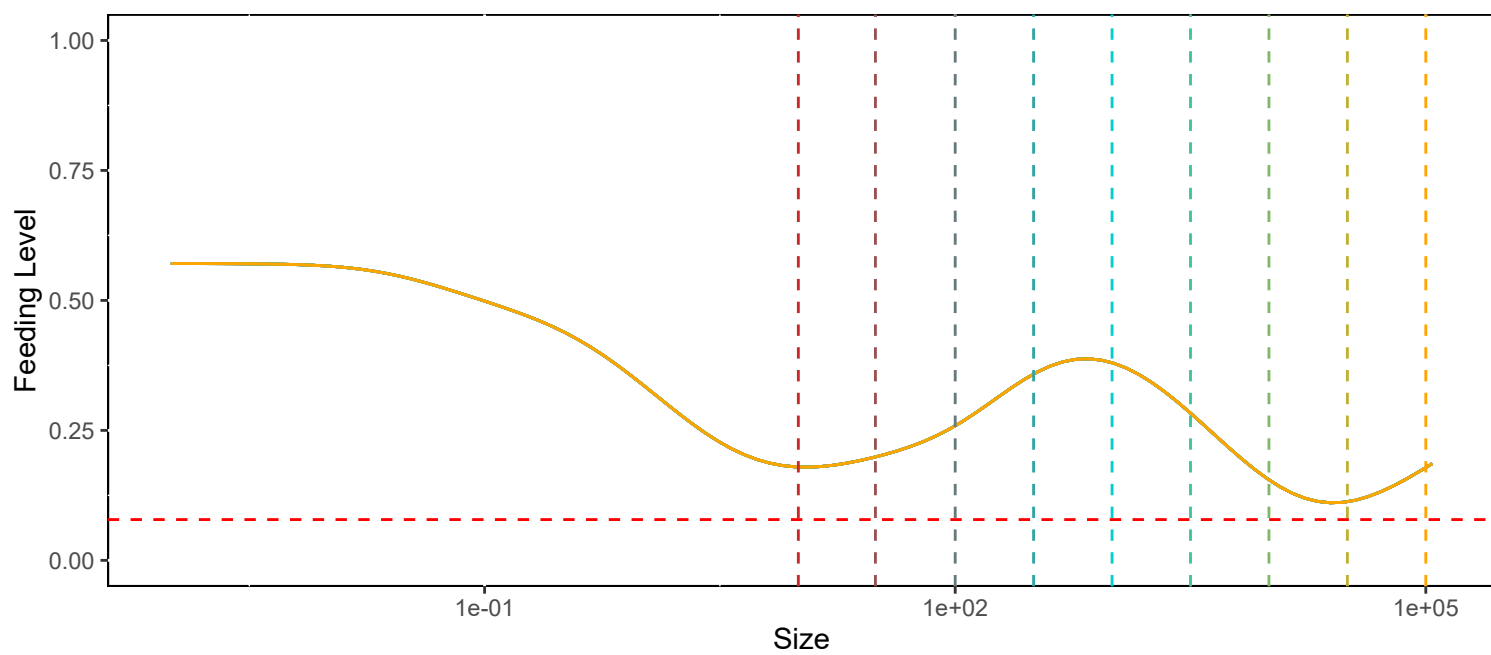

b)

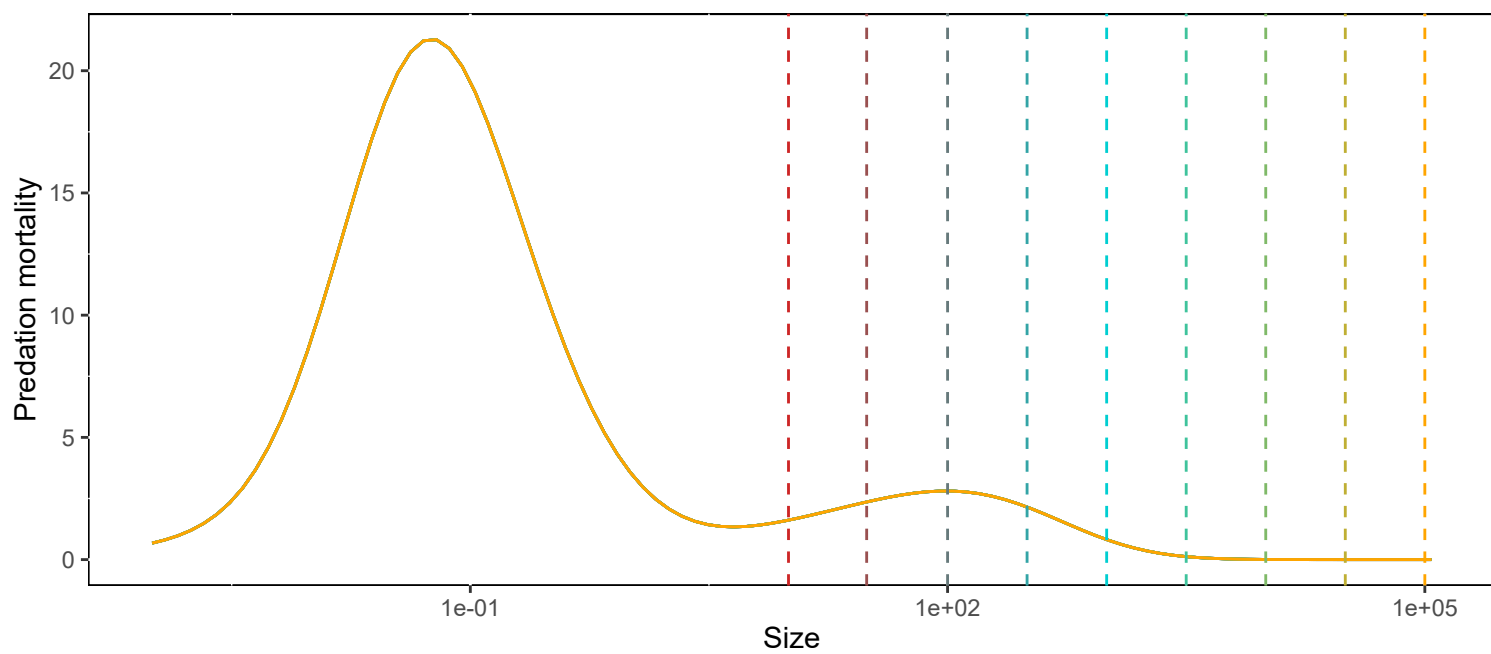

Species 1 2 3 4 5 6 7 8 9

Supplement: Supplementary file 11 — Fig S11 [file ECE3-10-14033-s011.pdf]
