# Supplementary material for: Interacting forces of predation and fishing affect species’ maturation size
Source: Ecol Evol. 2020 Dec 5;10(24):14033–51. doi: 10.1002/ece3.6995 (PMC7771143; doi:10.1002/ece3.6995)
Supplement: Supplementary file 12 — Tbl S1 [file ECE3-10-14033-s012.pdf]

Model selection table. df is degrees of freedom; logLik is likelihood; AICc is Akaike information criterion; delta is change in AICc. fsh - fishing, prd - predation, sp - species (all treated as factors). The best model is on the top row and includes all factors and their interactions. The second best model (second row) does not include the three-way interaction.

	(Intercept)	fsh	prd	sp	fsh:prd	fsh:sp	prd:sp	f:p:s	df	logLik	AICc	delta
128	-3.2	+	+	+	+	+	+	+	37	-147.38	377.49	0
64	-3.12	+	+	+	+	+	+		29	-158.61	380.5	3.01
56	-3.19	+	+	+		+	+		28	-164.83	390.57	13.08
48	-3.29	+	+	+	+		+		21	-190.11	424.96	47.47
40	-3.36	+	+	+			+		20	-195.35	433.18	55.69
39	-3.13		+	+			+		19	-241.79	523.82	146.33
32	-3.02	+	+	+	+	+			21	-274.09	592.91	215.42
24	-3.09	+	+	+		+			20	-277.39	597.26	219.77
16	-3.19	+	+	+	+				13	-291.36	609.77	232.28
8	-3.26	+	+	+					12	-294.36	613.62	236.13
