# Supplementary material for: Interacting forces of predation and fishing affect species’ maturation size
Source: Ecol Evol. 2020 Dec 5;10(24):14033–51. doi: 10.1002/ece3.6995 (PMC7771143; doi:10.1002/ece3.6995)
Supplement: Supplementary file 13 — Tbl S2 [file ECE3-10-14033-s013.pdf]

ANOVA table for the 3-way model $log(\eta)$ Species*Predation*Fishing. Partial effects sizes for each term are also reported (pes). Model is the first line of table S1

	Df	Sum Sq	Mean Sq	F value	Pr(>F)	pes
species	8	190.27	23.78	161.22	0	0.79
predation	1	42.14	42.14	285.65	0	0.46
fisheries	1	18.36	18.36	124.5	0	0.27
species:predation	8	45.75	5.72	38.77	0	0.48
species:fisheries	8	9.73	1.21	8.24	0	0.16
predation:fisheries	1	1.78	1.78	12.12	0	0.03
species:predation:fisheries	8	3.07	0.38	2.6	0.01	0.06
Residuals	324	47.79	0.14	NA	NA	NA
