# Supplementary material for: Interacting forces of predation and fishing affect species’ maturation size
Source: Ecol Evol. 2020 Dec 5;10(24):14033–51. doi: 10.1002/ece3.6995 (PMC7771143; doi:10.1002/ece3.6995)
Supplement: Supplementary file 14 — Tbl S3 [file ECE3-10-14033-s014.pdf]

Empirical size data and their references used in Fig.6

Species	Maturation size	Asymptotic size	References
Cod	3337.375666	19428.63866	Jennings et al. 1998
Haddock	334.656743	3117.98674	Jennings et al. 1998
Herring	111.102066	221.41796	Goodwin et al. 2006
Norway pout	46.924711	86.11638	Jennings et al. 1998
Plaice	203.236035	1496.00467	Jennings et al. 1998
Saithe	1459.741393	45627.15377	Jennings et al. 1998
Sandeel	3.563648	34.52603	Goodwin et al. 2006
Sole	150.154812	684.44291	Jennings et al. 1998
Sprat	15.674781	32.24996	Bailey 1980
Whiting	60.689	612.10952	Jennings et al. 1998
Dab	21.712738	211.24228	Rijnsdorp et al. 1992
Grey Gurnard	103.313363	886.01239	Fishbase
Anoplopoma fimbria	2243.365824	3388.987552	Olsson and Gislason, 2016
Anoplopoma fimbria	2495.410527	2591.429307	Olsson and Gislason, 2016
Sebastes crameri	839.2709096	1623.941784	Olsson and Gislason, 2016
Sebastes elongatus	114.0872975	727.4969882	Olsson and Gislason, 2016
Sebastes flavidus	1159.479145	2013.986983	Olsson and Gislason, 2016
Clupea harengus	144.4055108	234.7223797	Olsson and Gislason, 2016
Clupea harengus	152.5260593	306.5630249	Olsson and Gislason, 2016
Clupea harengus	127.5720137	219.7998486	Olsson and Gislason, 2016
Clupea harengus	215.6687028	367.248827	Olsson and Gislason, 2016
Clupea harengus	122.5440299	245.7383233	Olsson and Gislason, 2016
Clupea harengus	236.954886	382.5939548	Olsson and Gislason, 2016
Clupea harengus	240.5513444	330.4041016	Olsson and Gislason, 2016
Clupea pallasi	120.1927067	215.8984944	Olsson and Gislason, 2016
Clupea pallasi	173.2437888	453.506854	Olsson and Gislason, 2016
Clupea pallasi	66.6359996	215.8984944	Olsson and Gislason, 2016
Gadus macrocephalus	1448.78265	11769.19016	Olsson and Gislason, 2016
Gadus morhua	5825.230626	22718.35315	Olsson and Gislason, 2016
Gadus morhua	3472.860386	20086.52614	Olsson and Gislason, 2016
Gadus morhua	2288.576711	10111.50945	Olsson and Gislason, 2016
Gadus morhua	2021.927647	3262.315803	Olsson and Gislason, 2016
Gadus morhua	1190.137173	1645.589879	Olsson and Gislason, 2016
Gadus morhua	1768.349977	14147.75821	Olsson and Gislason, 2016
Melanogrammus aeglefinus	293.170736	3279.323679	Olsson and Gislason, 2016
Melanogrammus aeglefinus	698.1900651	2630.904545	Olsson and Gislason, 2016
Melanogrammus aeglefinus	707.9575712	3834.294493	Olsson and Gislason, 2016
Merlangius merlangus	179.256487	1495.422983	Olsson and Gislason, 2016
Merlangius merlangus	62.34150354	620.9007471	Olsson and Gislason, 2016
Theragra chalcogramma	294.78	3014.60454	Olsson and Gislason, 2016
Theragra chalcogramma	379.8975	730.02	Olsson and Gislason, 2016
Theragra chalcogramma	555.66	1509.22542	Olsson and Gislason, 2016
Trisopterus esmarkii	42.4700496	76.1849616	Olsson and Gislason, 2016
Hippoglossoides platessoides	878.5564359	5363.615599	Olsson and Gislason, 2016
Pleuronectes ferruginae	231.7439325	623.8158895	Olsson and Gislason, 2016
Pleuronectes ferruginae	141.712941	706.969943	Olsson and Gislason, 2016
Pleuronectes platessa	251.4994817	2287.908563	Olsson and Gislason, 2016
Pleuronectes platessa	188.4555558	1049.062573	Olsson and Gislason, 2016
Pleuronectes platessa	197.3690262	1749.712324	Olsson and Gislason, 2016
Solea solea	134.6253253	1233.978732	Olsson and Gislason, 2016
Solea solea	145.1377053	591.8365986	Olsson and Gislason, 2016
Carcharhinus brevipinna	57578.96575	178213.8916	Olsson and Gislason, 2016
Carcharhinus falciformis	79173.44259	181720.6787	Olsson and Gislason, 2016
Carcharhinus isodon	8814.472849	18111.37362	Olsson and Gislason, 2016
Carcharhinus limbatus	15063.79472	26326.34765	Olsson and Gislason, 2016
Carcharhinus sorrah	8153.456825	22868.00295	Olsson and Gislason, 2016
Carcharhinus sorrah	4704.774274	11108.36879	Olsson and Gislason, 2016
Carcharhinus tilstoni	11054.41718	48226.29158	Olsson and Gislason, 2016
Galeorhinus galeus	13556.40429	23279.53846	Olsson and Gislason, 2016
Mustelus antarcticus	5515.412778	37633.86675	Olsson and Gislason, 2016
Mustelus antarcticus	5840.80978	48712.8062	Olsson and Gislason, 2016
Prionace glauca	203867.6355	371380.9422	Olsson and Gislason, 2016
Menidia menidia	2.3	9.3	Conover and Munch, 2002
Menidia menidia	3.8	10	Conover and Munch, 2002
Menidia menidia	5.2	10.5	Conover and Munch, 2002
Poecilia reticulata	0.0761	0.590746429	Reznik et al., 1997
Poecilia reticulata	0.0675	0.590746429	Reznik et al., 1997
Poecilia reticulata	0.1892	3.602635961	Reznik et al., 1997
Poecilia reticulata	0.1623	3.602635961	Reznik et al., 1997
Danio rerio	0.0749	0.2134	Ulloa et al., 2011
